# Chondrocytes Transdifferentiate into Osteoblasts in Endochondral Bone during Development, Postnatal Growth and Fracture Healing in Mice

**DOI:** 10.1371/journal.pgen.1004820

**Published:** 2014-12-04

**Authors:** Xin Zhou, Klaus von der Mark, Stephen Henry, William Norton, Henry Adams, Benoit de Crombrugghe

**Affiliations:** 1 Department of Genetics, The University of Texas MD Anderson Cancer Center, Houston, Texas, United States of America; 2 Department of Experimental Medicine 1, Nikolaus-Fiebiger-Center of Molecular Medicine, University of Erlangen-Nuremberg, Erlangen, Germany; 3 Department of Veterinary Medicine & Surgery, The University of Texas MD Anderson Cancer Center, Houston, Texas, United States of America; Children's Hospital Boston and Harvard Medical School, Howard Hughes Medical Institute, United States of America

## Abstract

One of the crucial steps in endochondral bone formation is the replacement of a cartilage matrix produced by chondrocytes with bone trabeculae made by osteoblasts. However, the precise sources of osteoblasts responsible for trabecular bone formation have not been fully defined. To investigate whether cells derived from hypertrophic chondrocytes contribute to the osteoblast pool in trabecular bones, we genetically labeled either hypertrophic chondrocytes by *Col10a1-Cre* or chondrocytes by tamoxifen-induced *Agc1-CreERT2* using *EGFP*, *LacZ or Tomato* expression. Both Cre drivers were specifically active in chondrocytic cells and not in perichondrium, in periosteum or in any of the osteoblast lineage cells. These *in vivo* experiments allowed us to follow the fate of cells labeled in *Col10a1-Cre* or *Agc1-CreERT2* -expressing chondrocytes. After the labeling of chondrocytes, both during prenatal development and after birth, abundant labeled non-chondrocytic cells were present in the primary spongiosa. These cells were distributed throughout trabeculae surfaces and later were present in the endosteum, and embedded within the bone matrix. Co-expression studies using osteoblast markers indicated that a proportion of the non-chondrocytic cells derived from chondrocytes labeled by *Col10a1-Cre* or by *Agc1-CreERT2* were functional osteoblasts. Hence, our results show that both chondrocytes prior to initial ossification and growth plate chondrocytes before or after birth have the capacity to undergo transdifferentiation to become osteoblasts. The osteoblasts derived from *Col10a1*-expressing hypertrophic chondrocytes represent about sixty percent of all mature osteoblasts in endochondral bones of one month old mice. A similar process of chondrocyte to osteoblast transdifferentiation was involved during bone fracture healing in adult mice. Thus, in addition to cells in the periosteum chondrocytes represent a major source of osteoblasts contributing to endochondral bone formation *in vivo*.

## Author Summary

During endochondral bone formation, which is responsible for the generation of most bones in mammals and many other species, osteoblasts deposit a bone-specific matrix on the surface of cartilage scaffolds made by chondrocytes and hypertrophic chondrocytes. It has long been thought that the terminally differentiated chondrocytes in this cartilage scaffold undergo cell death. Here we demonstrate that chondrocytes can transdifferentiate into osteoblasts and that these transdifferentiated osteoblasts represent a substantial fraction of the bone forming cells in mice, We also provide evidence that chondrocytes can transdifferentiate into osteoblasts during bone fracture repair, a process similar to endochondral bone formation.

## Introduction

Long bones, ribs, vertebrae, and other parts of the vertebrate skeleton are formed through a precisely synchronized process known as *endochondral ossification*. The highly complex endochondral bone tissue, which is generated through a cartilage intermediate, consists of multiple types of cells, including mesenchymal-derived chondrocytes, osteoblasts and osteocytes, as well as osteoclasts and bone marrow cells, which have a hematopoietic origin [1].

Endochondral bone is made of an outer compact bone (cortex) and an inner spongy bone tissue within the bone marrow cavity. The conversion from the nonvascular cartilage template to fully mineralized endochondral bones proceeds in distinct and closely coupled steps [2]. The first step is initiated when chondrocytes in the center of the cartilage models undergo hypertrophic differentiation and cells in the perichondrium surrounding the hypertrophic zone differentiate into osteoblasts to form the interim bone cortex (bone collar). Concurrently, the initial vascular invasion occurs in the same region importing blood vessel-associated pericytes, osteoclasts and progenitor cells in the circulating blood. Immediately following the onset of bone collar formation, hypertrophic chondrocytes and the mineralized cartilage matrix in the center of the cartilage template are replaced by a highly vascularized trabecular bone tissue as well as bone marrow. Bone trabeculae in the primary spongiosa are formed by deposition of osteoid by osteoblasts on the surface of calcified cartilage spicules.

Until recently, the precise origins of the trabeculae-producing osteoblasts had remained largely undefined. A lineage tracing study using tamoxifen-inducible CreER transgenic mice harboring a *ROSA26R* conditional allele showed that *Osx*-expressing immature osteoblast precursors, labeled in the perichondrium before the development of the primary ossification center, were able to migrate into the cartilage along with blood vessels and were responsible, at least in part, for trabeculae formation [3], [4].

Besides perichondrium cells, several other cellular sources have also been examined as potential candidates to account for the formation of the primary spongiosa, including pericytes associated with the invading blood vessels, bone marrow progenitor cells and hypertrophic chondrocytes [4].

During endochondral ossification, terminally differentiated hypertrophic chondrocytes are eventually completely removed from the initial cartilage template or growth plates. Some of these chondrocytes have been shown to be eliminated through either apoptosis or autophagy (type II programmed cell death) [5], [6], [7], [8]. However, the fate of the hypertrophic chondrocytes not accounted for by either of these processes of cell death continues to be debated. It has been shown that hypertrophic chondrocytes also express osteoblast markers, such as Alkaline Phosphatase (ALPL), Osteonectin (SPARC), Osteocalcin (BGLAP), Osteopontin (SPP1) and Bone sialoprotein (IBSP), implicating potential complex functions of these cells [5], [6]. Indeed, a number of studies have reported that in cell cultures containing ascorbic acid or in organ cultures, hypertrophic chondrocytes, instead of becoming extinct, resume cell proliferation and undergo asymmetric cell division, giving rise to cells with morphological and phenotypic characteristics of osteoblasts capable of producing a mineralized bone matrix in vitro [6], [7], [8], [9]. In addition, histological experiments suggested that in embryonic chicks, long bone chondrocytes differentiated to bone-forming cells and deposited bone matrix inside their lacunae [10], further promoting the notion of a direct role of hypertrophic chondrocytes in trabecular bone formation. However, most of the evidence supporting transdifferentiation was based on either histological observations during skeletal development or on *in vitro* studies [11]. Overall, it was suggested that these studies were not fully conclusive (3). The result of an earlier *ex vivo* experiment that modified mouse embryonic limb tissue was consistent with a hypothetical transdifferentiation of chondrocytes into osteoblasts but the cells were not further characterized [12]. However, the conclusions of two more recent lineage tracing studies did not support a contribution of mature chondrocytes to the osteoblast/osteocyte pool in the central metaphyseal regions below the growth cartilage [3], [13].

Mature osteoblasts develop from *Runx2*-expressing osteoblast precursors that are derived from mesenchymal progenitors [14], [15]. Osterix (Osx, Sp7) is a key transcription factor required for the full differentiation of *Runx2*-expressing osteoblast precursors into mature osteoblasts and osteocytes [16]. *Osx* is expressed in osteoblasts and osteocytes but also, at a lower level, in prehypertrophic and hypertrophic chondrocytes and in bone marrow mesenchymal progenitor cells during and after embryonic development [17]. Inactivation of Osx during and after embryonic development completely arrested osteoblast differentiation and bone formation [16], [17].

The purpose of this study was to examine whether hypertrophic chondrocytes may acquire an osteogenic fate *in vivo*. We specifically labeled either hypertrophic chondrocytes with a Cre recombinase driven by a Collagen 10 BAC transgene (*Col10a1-Cre*) [18] or chondrocytes with an inducible Cre recombinase, the DNA of which was inserted by knock-in in the 3′ UTR of the Aggrecan gene (*Agc1-CreERT2*) [19]. Labeling occurred with either *EGFP* [20], *LacZ* [21] or *Tomat*o expression [22]. *LacZ* and *Tomato* expression were from conditional alleles in the ROSA locus. The *EGFP* DNA preceded by a *LoxP* site was inserted 3′ to the poly-A site of *Osx* whereas in this allele the other *LoxP* site was placed in the first intron of the *Osx* gene [23]. In mice harboring this allele, high expression of *EGFP* occurs only in *Osx*-expressing cells after the *LoxP* sites recombine (S1A Figure) [23]. Neither of the two Cres was expressed in the perichondrium or the periosteum of endochondral bones [18], [19]. Upon recombination, ROSA26R reporter mouse expresses secreted β-galactosidase (LacZ), ROSA-Tomato reporter mouse expresses cytoplasmic tandem dimer Tomato, and Osx floxed mouse expresses cytoplasmic EGFP. Whereas labeling of mature chondrocytes in mice harboring *Col10a1-Cre* occurred constitutively once its expression begun and persisted as long as the *Col10a1* promoter remained active, the timing of labeling of chondrocytes by *Agc1-CreERT2* was controlled by the administration of tamoxifen and this labeling period persisted for a short period. One advantage of the *Osx/EGFP* allele in cell fate experiments was that if one would detect non-chondrocytic cells expressing EGFP, these cells would likely be osteoblast lineage cells [16], [23].

Our data show that labeled non-chondrocytic cells appeared in the primary spongiosa of *Col10a1-Cre* or of tamoxifen activated *Agc1-CreERT2* embryos and mice. In the case of *Col10a1-Cre* embryos and in *Agc1-CreERT2* embryos treated with tamoxifen earlier than E14.5, these non-chondrocytic reporter^+^ cells started to appear at the onset of primary ossification. Later they were found throughout the primary ossification centers and subsequently in the endosteum and within the bone matrix. Their appearance could also be induced in the primary spongiosa postnatally. Many of these cells expressed the mature osteoblast marker Osteocalcin and exhibited osteoblast-specific *Col1a1*-promoter activity. Likewise, in tamoxifen treated *Agc1-CreERT2* mice chondrocyte-derived reporter^+^ non-chondrocytic cells were present in the repair callus of fractured tibiae. Later these reporter^+^ cells, which were associated with the ossified bone matrix in the calluses, also displayed *Col1a1*-promoter activity. Our results provide *in vivo* evidence that chondrocytes, both in cartilage primordium and in established growth plates, as well as chondrocytes in bone repair calluses, have the capacity to transdifferentiate into osteoblasts and represent a major source of osteoblasts in endochondral bones.

## Results

### Abundance of *Col10a1-Cre* induced reporter^+^ cells throughout the primary spongiosa of *Col10a1-Cre* reporter-containing embryos and mice

In *Col10a1-Cre* transgenic mice, Cre recombinase activity was previously detected specifically in all hypertrophic chondrocytes starting from E13.5 throughout endochondral skeletal development and into the postnatal stage [18]. Here we further confirmed that in the femurs and tibias of E15.5 *Col10a1-Cre; ROSA26R* mice, only hypertrophic chondrocytes, not cells in the perichondrium and periosteum, were positive for LacZ (S1B-a, b Figure.), indicating that Cre activity driven by the *Col10a1* regulatory elements occurred specifically in hypertrophic chondrocytes in these *Col10a1-Cre* transgenic mice. This was also confirmed by in situ hybridization of *Col10a1* and *Cre* mRNA which was only observed in the hypertrophic zone (S1C Figure). To test the hypothesis that some of the *Col10a1*-expressing hypertrophic chondrocytes might transdifferentiate into osteoblasts, we crossed *Col10a1-Cre* with *Osx ^flox/flox^* mice to generate *Col10a1-Cre; Osx ^flox/+^* embryos. In these embryos *EGFP* expression labels *Osx*-expressing cells, which either expressed *Col10a1* or were derived from *Col10a1*-expressing cells (Figure S1A).

**Figure 1:**
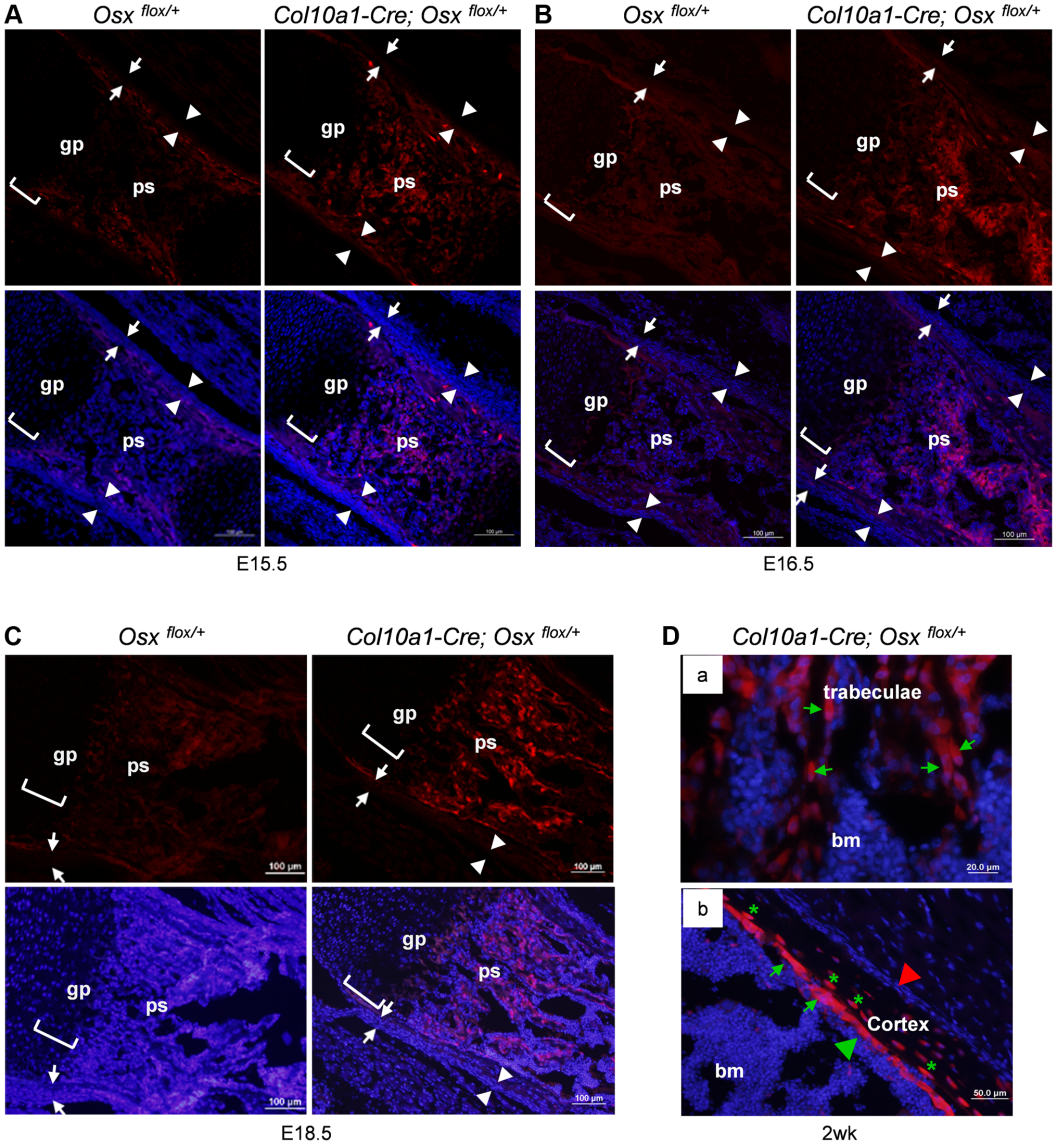
Presence of abundant EGFP^+^ (*Osx^−/+^*) cells throughout the primary spongiosa of *Col10a1-Cre; Osx^flox/+^* embryos and mice. Anti-EGFP immunofluorescence (IF) showed that EGFP*^+^* (*Osx^−/+^*) cells (red) were found in the primary spongiosa of E15.5 (A), E16.5 (B) and E18.5 (C) *Col10a1-Cre; Osx ^flox/+^* femurs (right panels), but not in the *Osx ^flox/+^* control embryos (left panels). The controls showed auto-fluorescence produced by mineralized tissue and bone marrow cells, but no EGFP*^+^* cells. Upper panels: anti-EGFP (red); Lower panels: anti-EGFP and DAPI (blue). There were virtually no EGFP^+^ cells in the perichondrium (between white arrows) or periosteum (between white arrowheads) of the *Col10a1-Cre; Osx ^flox/+^* embryos. D: In the femurs of 2-week-old *Col10a1-Cre; Osx^flox/+^* mice, EGFP^+^ (*Osx^−/+^*) cells (green arrows) were found throughout the trabeculae (panel a), on the endosteum surface (panel b), and embedded within the cortex (green asterisks). Red arrowhead: periosteum; Green arrowhead: endosteum; gp: growth plate (white brackets); ps: primary spongiosa; bm: bone marrow. In the *Osx ^flox/+^* control embryos, weak EGFP^+^ cells were present in the perichondrium and periosteum area (also in Fig. 3C), indicating that there was a low level read through of EGFP independent of Cre-mediated excision of floxed *Osx* allele.

Immunofluorescence (IF) with anti-EGFP showed that in the femurs of *Col10a1-Cre; Osx ^flox/+^* embryos, abundant EGFP-positive (EGFP^+^) cells were present throughout the primary ossification centers (Fig. 1), where only very few, if any, *Col10a1*- or *Cre*-expressing cells were detected (S1C Figure). The appearance of these EGFP^+^ cells was concurrent with the onset of primary ossification at E15.5 (Fig. 1A). These EGFP^+^ cells continued to be present in the primary spongiosa at E16.5 (Fig. 1B), E18.5 (Fig. 1C) to after birth (Fig. 1D). The EGFP expression levels of these cells increased from E15.5 to E18.5 in parallel to the increasing levels of *Osx* expression in the same regions. In the 2-week-old *Col10a1-Cre; Osx ^flox/+^* mice, EGFP^+^ cells were found throughout the trabecular surfaces, also lining the endosteum of the distal half of the femur, and even embedded within the bone matrix of the cortical bone and trabeculae (Figure S1D, Fig. 1D).

Such EGFP^+^ non-chondrocytic cells were completely absent in intramembranous skeletal elements such as the calvariae of E18.5 *Col10a1-Cre; Osx ^flox/+^* embryos (Figure S1E). In addition to its expression in hypertrophic chondrocytes and osteoblasts in the primary spongiosa, *Osx* was also highly expressed in periosteum and perichondrium [16]. However, EGFP^+^ cells were only detected in primary ossification centers and, as to be expected at lower levels in the hypertrophic zone, not in periosteum and perichondrium. This indicated that these EGFP^+^ non-chondrocytic cells were cells in which the *Osx* floxed allele was recombined by *Col10a1-Cre* and that these cells were unlikely to be derived from periosteum or perichondrium cells (Fig. 1). Staining of EGFP^+^ hypertrophic chondrocytes was not strong enough to be clearly shown due to strong autofluorescence from mineralized cartilage and bone matrix and from blood cells in the bone marrow.

**Figure 2:**
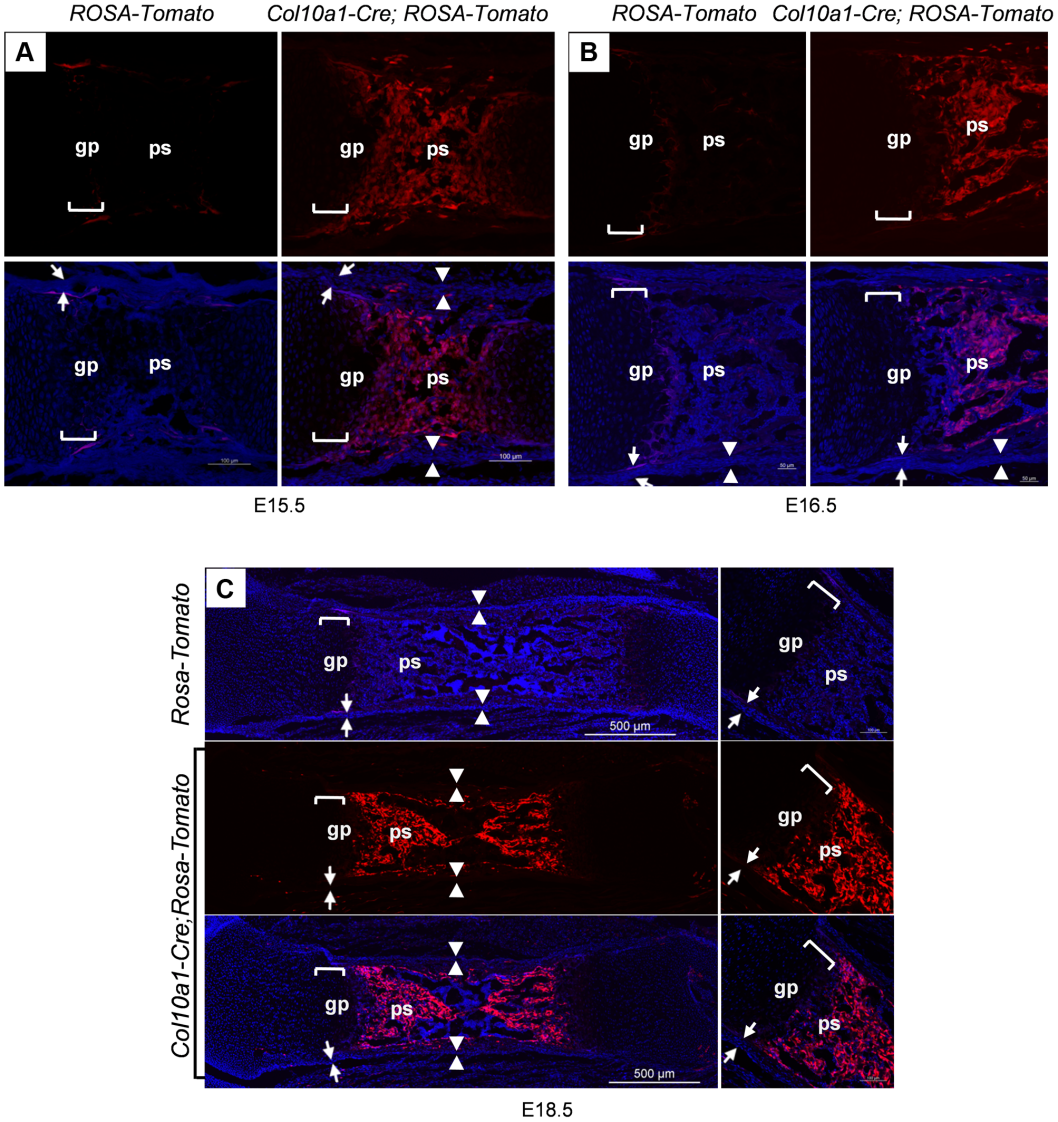
Presence of abundant Tomato^+^ cells throughout the primary spongiosa of *Col10a1-Cre; ROSA-tdTomato* embryos. *Col10a1-Cre;ROSA-tdTomato* embryos were generated to verify the observations made in the *Col10a1-Cre;Osx ^flox/+^* embryos. Abundant Tomato^+^ non-chondrocytic cells (red) were present throughout the primary spongiosa in femurs of E15.5 (A), E16.5 (B) and E18.5 (C) *Col10a1-Cre;ROSA-tdTomato* embryos (right panels). Tomato^+^ non-chondrocytic cells were completely absent from the *ROSA-tdTomato* control embryos in all stages evaluated (left panels in A and B, upper panel in C). Upper panels in A and B and middle panel in C: red channel; Lower panels: red and blue channels. Right panels in C: magnified images. There were virtually no Tomato^+^ cells within the perichondrium (between white arrows) or periosteum (between white arrowheads) of the *Col10a1-Cre; ROSA-tdTomato* embryos. gp: growth plate (white brackets); ps: primary spongiosa; bm: bone marrow.

Similarly, *Col10a1-Cre* induced Tomato^+^ cells were found throughout the primary spongiosa of *Col10a1-Cre; ROSA-tdTomato* embryos (Fig. 2A, B and C) in the same pattern as in the *Col10a1-Cre; Osx ^flox/+^* embryos. This result confirmed our observation that cells derived from *Col10a1-Cre* expressing hypertrophic chondrocytes were indeed present in the primary spongiosa. In the femurs of 6-month-old *Col10a1-Cre; ROSA-tdTomato*;*Osx ^flox/+^* mice, Tomato^+^ cells continued to be present, however, the number of these cells on the endosteum surface was reduced and these cells were distributed more evenly from endosteum to periosteum within the cortex (S1F Figure). By 8 months, there were almost no Tomato^+^ cells present in the primary spongiosa. The Tomato^+^ cells were distributed mostly within the bone matrix or in the growth plate. Besides a few strong Tomato^+^ chondrocytes in the growth plate, the fluorescence intensity of a majority of Tomato^+^ cells were much weaker than in the younger mice (Figure S1G).

### The non-chondrocytic reporter^+^ cells in the primary spongiosa of *Col10a1-Cre* or tamoxifen-treated *Agc1-CreERT2* embryos and mice are derived from mature chondrocytes

To verify the presence in the primary spongiosa of reporter^+^ cells that were labeled by *Col10a1-Cre*, and to further delineate the origin of these cells we generated inducible *Agc1-CreERT2; ROSA26R* mouse models. In *Agc1-CreERT2; ROSA26R* mice, tamoxifen-induced Cre recombinase activity occurred efficiently in all chondrocytes including hypertrophic chondrocytes during and after development [19]. In the limbs of E14.5 *Agc1-CreERT2; ROSA26R* embryos retrieved from a female treated with tamoxifen at E13.5, the majority of chondrocytes, including pre- and hypertrophic chondrocytes, were positive for LacZ, while essentially no LacZ^+^ cells were found in the perichondrium, indicating that the *Agc1-CreERT*2-mediated recombination took place specifically in chondrocytes, not in perichondrium cells (Figure S2A-a, a′, b, b′).

As described in our previous report [19], E12.5 *Agc1-CreERT2; ROSA26R* embryos were negative for LacZ when treated with tamoxifen at E11.5 (Table S1), while E13.5 *Agc1-CreERT2; ROSA26R* embryos were positive for LacZ when treated with tamoxifen at E12.5. Experiments detailed in the next paragraph imply that it took between 9 and 18 hours after tamoxifen administration to accumulate enough β–galactosidase in order to obtain a clear readout in *Agc1-CreERT2; ROSA26R* embryos. Hence it is likely that E12.5 was the earliest embryonic stage for *Agc1-CreERT2* activity in the femur of these embryos. To determine the length of time that tamoxifen remains capable of activating *Agc1-CreERT2* after intraperitoneal injection, E13.5 *Agc1-CreERT2;ROSA26R* embryos were retrieved from pregnant females treated with tamoxifen at either E8.5, E9.5 or E11. E13.5 *Agc1-CreERT2;ROSA26R* embryos treated with tamoxifen at E11 were positive for LacZ staining, while E13.5 *Agc1-CreERT2; ROSA26R* embryos treated with tamoxifen at E8.5 or E9.5 were negative for LacZ, indicating that tamoxifen has a Cre activation window of three days or less after injection (Figure S2B and Table S1).

**Figure 3:**
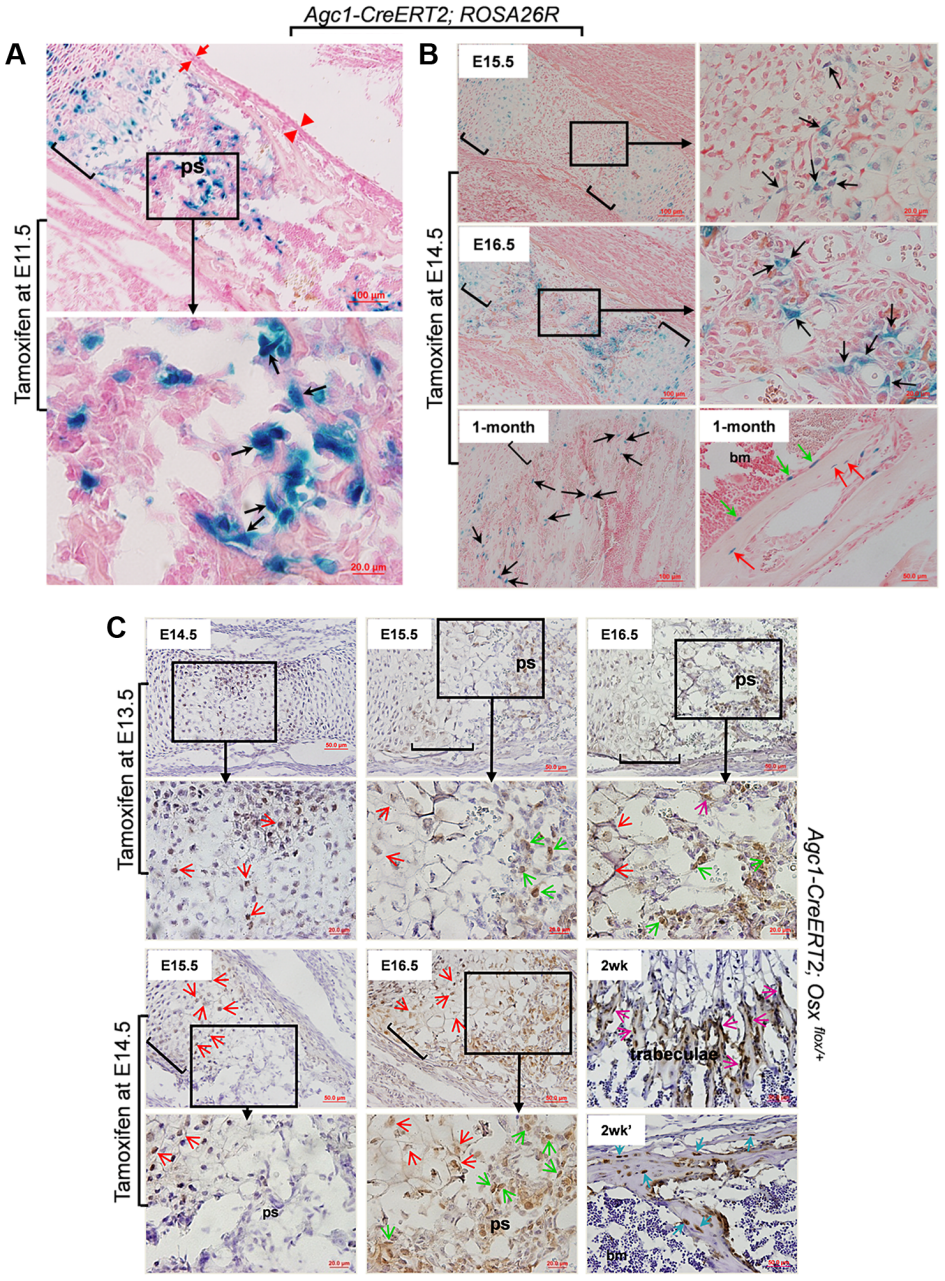
The non-chondrocytic reporter^+^ cells in the primary spongiosa of tamoxifen-treated *Agc1-CreERT2* embryos and mice are derived from mature chondrocytes. A: The LacZ stained femur section of E16.5 *Agc1-CreERT2; ROSA26R* embryo treated with tamoxifen at E11.5. The black arrows indicate the non-chondrocytic LacZ^+^ cells in the primary spongiosa. Black brackets: hypertrophic zone. No LacZ^+^ cells were detected within the perichondrium (red arrows) and periosteum (red arrowheads). B: LacZ staining of *Agc1-CreERT2; ROSA26R* femurs treated with tamoxifen at E14.5 and euthanized 1 and 2 days after injections or at 1 month postnatally. At E15.5, LacZ^+^ non-chondrocytic cells (black arrows) were only present right beneath the growth plates, while at E16.5, the number of LacZ^+^ non-chondrocytic cells was increased. At 1 month, LacZ^+^ cells were still present on the trabecular surfaces (black arrows), endosteum (green arrows) and within bone matrix (red arrows). Black brackets: hypertrophic zone. C: Anti-EGFP Immunohistochemical analysis (IHC) of *Agc1-CreERT2; Osx ^flox/+^* mice treated with tamoxifen at E13.5 or E14.5 and euthanized 1, 2 and 3 days after injections or 2 weeks postnatally. The data show that there were abundant non-chondrocyte EGFP^+^ (*Osx^−/+^*) cells in the primary spongiosa of the femur of E15.5 *Agc1-CreERT2; Osx ^flox/+^* embryos treated with tamoxifen at E13.5. However, there were almost no non-chondrocyte EGFP^+^ cells in the primary spongiosa of E15.5 *Agc1-CreERT2; Osx ^flox/+^* embryos treated with tamoxifen at E14.5. Red arrows: EGFP^+^ (*Osx^−/+^*) hypertrophic chondrocytes. Green arrows: preosteoblast-like EGFP^+^ (*Osx^−/+^*) cells. Purple arrows: mature osteoblast-like EGFP^+^ (*Osx^−/+^*) cells. Blue arrows: EGFP^+^ (*Osx^−/+^*) osteocytes. Black brackets: hypertrophic zone; ps: primary spongiosa.

To further assess the dynamics of the LacZ labeling after tamoxifen injection, we administered tamoxifen to pregnant females of E15.5 *Agc1-CreERT2; ROSA26R* embryos and followed the appearance of LacZ^+^ cells in the femur of these embryos at successive times post-injection. At 9 hours post-injection weak LacZ^+^ chondrocytes including hypertrophic chondrocytes started to appear, at 18 hours many chondrocytes and hypertrophic chondrocytes were strongly positive for LacZ and very few LacZ^+^ cells were detected in the primary spongiosa. At 24 hours post injection significantly more cells in the primary spongiosa were positive for LacZ (Figure S2C). This suggested that there was a sequential appearance of LacZ staining first in chondrocytes between 9 and 18 hours and then in cells of the primary spongiosa after tamoxifen injection at E15.5 the time when ossification is initiated in femurs. We then injected tamoxifen in pregnant females of E11.5 *Agc1-CreERT2; ROSA26R* embryos four days earlier than the onset of primary ossification at E15.5 in the femur, a time when the Cre-inducing activity of tamoxifen was no longer retained in this tissue. These embryos were then sacrificed at E16.5. LacZ staining was observed not only in chondrocytes but also in non-chondrocytic cells in the primary spongiosa (Fig. 3A). This experiment largely ruled out that the presence of LacZ^+^ cells in the primary spongiosa might be due to transient ectopic expression of *Agc1-CreERT2* in nascent osteoblasts at E15.5 coupled with the tamoxifen induction of Cre recombinase in these cells. This experiment suggested that the labeled cells in the primary spongiosa were derived from chondrocytes, in which the ROSA locus was recombined by tamoxifen induction of Cre recombinase.

Also,in the limbs of E15.5 *Agc1-CreERT2; ROSA26R* embryos collected 1 day after tamoxifen injection at E14.5, only chondrocytes (including hypertrophic chondrocytes), and a few non-chondrocytic cells residing immediately below the proximal growth plate, were positive for LacZ, but no LacZ^+^ cells were present in the center of the marrow cavity in these embryos (Fig. 3B). By comparison, E15.5 *Col10a1-Cre;Osx ^flox/+^* and E15.5 *Col10a1-Cre; ROSA-tdTomato* embryos (Figs. 1A and 2A) showed more abundant labeled cells in the primary spongiosa because recombination and labeling of the hypertrophic chondrocytes occurred earlier. However, when embryos were retrieved at E16.5, 2 days after tamoxifen injection, non-chondrocytic LacZ^+^ cells were found from right under the growth plates gradually extending to the center of the marrow cavity (Fig. 3B). In the 1-month-old *Agc1-CreERT2; ROSA26R* mice, born to a female treated with tamoxifen at E14.5, LacZ^+^ cells were localized on the surfaces of trabeculae and the endosteum and embedded within the bone matrix (Fig. 3B). Together these results substantiated the notion that the tamoxifen-mediated appearance of non-chondrocytic LacZ^+^ cells in the primary spongiosa was specifically linked to the CreERT2 activity in chondrocytes and could be dissociated from the onset of primary ossification at E15,5.

To further validate our observations in *Agc1-CreERT2;ROSA26R* embryos and mice, we generated *Agc1-CreERT2; Osx ^flox/+^* embryos and mice, which were retrieved from tamoxifen-treated pregnant females or were the offspring of these female mice. In the femurs of E14.5 *Agc1-CreERT2; Osx ^flox/+^* embryos collected 1 day after tamoxifen treatment at E13.5 pre- and hypertrophic chondrocytes were positive for EGFP, consistent with the notion that the *Osx* floxed allele was recombined only in chondrocytes (Fig. 3C). In the femurs of E15.5 and E16.5 *Agc1-CreERT2; Osx ^flox/+^* embryos collected 2 and 3 days after tamoxifen treatment, not only were hypertrophic chondrocytes positive for EGFP but so were non-chondrocytic cells in priFmary ossification centers, similar to the findings in the *Col10a1-Cre; Osx ^flox/+^ embryos* (Fig. 1). However when we pulsed tamoxifen in E14.5 pregnant mice to tag the EGFP^+^ (*Osx^−/+^*) cells 1 day prior to the onset of primary ossification, almost no non-chondrocytic EGFP^+^ (*Osx^−/+^*) cells were detected in the primary ossification centers at E15.5, except a few at the proximal chondro-osseous interface (Fig. 3C). Whereas at E16.5, in addition to hypertrophic chondrocytes, abundant numbers of non-chondrocytic cells in the primary ossification centers were also positive for EGFP (Fig. 3C). Furthermore, in a 2-week-old *Agc1-CreERT2; Osx ^flox/+^* mice, an offspring of a female treated with tamoxifen at E14.5, the EGFP^+^ (*Osx^−/+^*) cells were lining the surfaces of trabeculae and endosteum. They were also found within the bone matrix (Fig. 3C), but were totally absent in calvariae (S2E Figure), similar to what was seen in the 2-week-old *Col10a1-Cre; Osx ^flox/+^* mice (Fig. 1D). Nevertheless, unlike the findings in the 2-week-old *Col10a1-Cre; Osx ^flox/+^* mice, in which Cre activity persisted in *Col10a1*-expressing cells, the hypertrophic chondrocytes were no longer positive for EGFP in the *Agc1-CreERT2; Osx ^flox/+^* mice. Together these experiments in *Agc1-CreERT2; Osx ^flox/+^* embryos indicated that the labeling of chondrocytes preceded the time of appearance of labeled non-chondrocytic cells in the primary spongiosa. The timing of these distinct processes was controlled by the time of tamoxifen injection in the pregnant females.

In a separate experiment a pregnant *Agc1-CreERT2;ROSA26R* female was treated with tamoxifen at E17.5 when growth plates in the femurs were fully established (Figure S2D) and her offspring was sacrificed two days after birth. In these pups LacZ^+^ non-chondrocytic cells were present in the primary spongiosa and were distributed in an dome-shaped area under the growth plate, suggesting that mature chondrocytes in the established growth plate continued to be an important source of these LacZ^+^ cells.

Collectively, the distinct temporal and spatial presence of reporter^+^ non-chondrocytic cells observed after recombination by either *Col10a1-Cre* or *Agc1-CreERT2* strongly suggested that these reporter^+^ non-chondrocytic cells were derived from mature chondrocytes.

### Chondrocytes have the ability to become bone-forming osteoblasts

**Figure 4:**
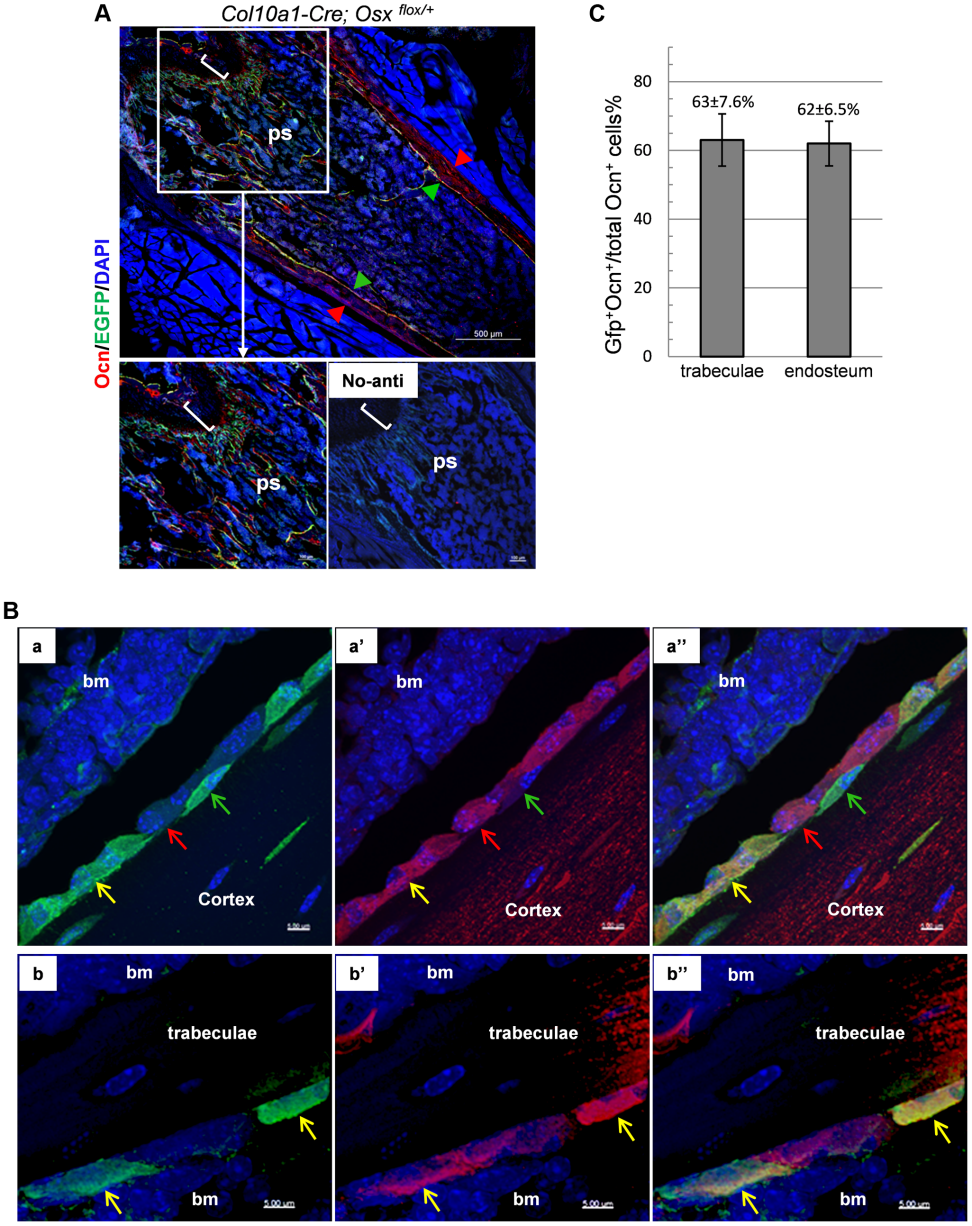
A double Immunofluorescence experiment revealed that the EGFP^+^ (Osx^−/+^) cells in the primary spongiosa and endosteum of *Col10a1-Cre; Osx^flox/+^* mice are mature osteoblasts. A, B and C: DIF experiment with anti-Ocn and anti-GFP using femur frozen sections of 1-month-old *Col10a1-Cre; Osx^flox/+^* mice. A: Lower right panel: no primary antibodies control. Upper and lower left panels: DIF experiment with anti-Ocn and anti-GFP. Anti-Ocn (red), anti-GFP (green) and DAPI (blue). White brackets indicate growth plate. ps: primary spongiosa. Red arrowhead: periosteum; Green arrowhead: endosteum. B: Magnified cell images of part of the upper panel in A. Panels a, a′ and a″: magnified cortical region. a: blue and green channels; a′: blue and red channels; a″: blue, green and red channels. Panels b, b′ and b″: magnified trabecular region. b: blue and green channels; b′: blue and red channels; b″: blue, green and red channels. Red arrows indicate Ocn^+^EGFP^−^ cells. Green arrows indicate EGFP^+^Ocn^−^ cells. Yellow arrows indicate Ocn^+^EGFP^+^ cells. Bm: bone marrow. Blue channel: 405 nm; Green channel: 488 nm; Red channel: 555 nm. C: Percent of Ocn^+^ mature osteoblasts which were derived from *Col10a1*-expressing mature chondrocytes in trabeculae and endosteum regions.

If the reporter^+^ cells, labeled at the time they existed as chondrocytes but later found in the trabecular region and in the endosteum, were functional osteoblasts, these cells should express typical osteoblast markers. To test this hypothesis we first performed double immunofluorescence (DIF) experiments with anti-EGFP and anti-Osteocalcin (Ocn) on the femur of 1-month-old *Col10a1-Cre;Osx ^flox/+^* mice. Non-chondrocytic EGFP^+^ cells represented cells derived from hypertrophic chondrocytes whereas cells positive for intracellular Osteocalcin (Ocn^+^) corresponded to mature osteoblasts. Hence cells that are positive for both markers (EGFP^+^Ocn^+^) are cells derived from chondrocytes that are functional osteoblasts. Shown in Fig. 4A, the EGFP^+^Ocn^+^ cells were indeed present in the femur of 1-month-old *Col10a1-Cre;Osx ^flox/+^* mice. High resolution confocal microscopy revealed that these Ocn^+^EGFP^+^ cells exhibited a characteristic mature osteoblast morphology (Fig. 4B). It was evident that not all Ocn^+^ cells on the bone surfaces were positive for EGFP, implying that there were other sources of osteoblasts besides hypertrophic chondrocytes. In addition, not all EGFP^+^ cells were positive for Ocn, since EGFP^+^ cells represented cells derived from mature chondrocytes at different stages of osteoblast differentiation. In the trabecular region, approximate 63% of Ocn^+^ cells were Ocn^+^EGFP^+^ cells, and about 62% Ocn^+^ endosteal cells were Ocn^+^EGFP^+^ cells (Fig. 4C and Table S2).

Consistent with this result, DIF with anti-EGFP and anti-Col1a1 antibodies revealed that the EGFP^+^ (*Osx^−/+^*) cells in the primary spongiosa were tightly associated with type I collagen in the femurs of E18.5 *Col10a1-Cre; Osx ^flox/+^* embryos (S3A Figure). Likewise, IHC with anti- BSP showed that many of the LacZ^+^ cells in the primary spongiosa were closely associated with BSP in the femur of E16.5 *Agc1-CreERT2; ROSA26R* treated with tamoxifen at E14.5 (Figure S3B).

**Figure 5:**
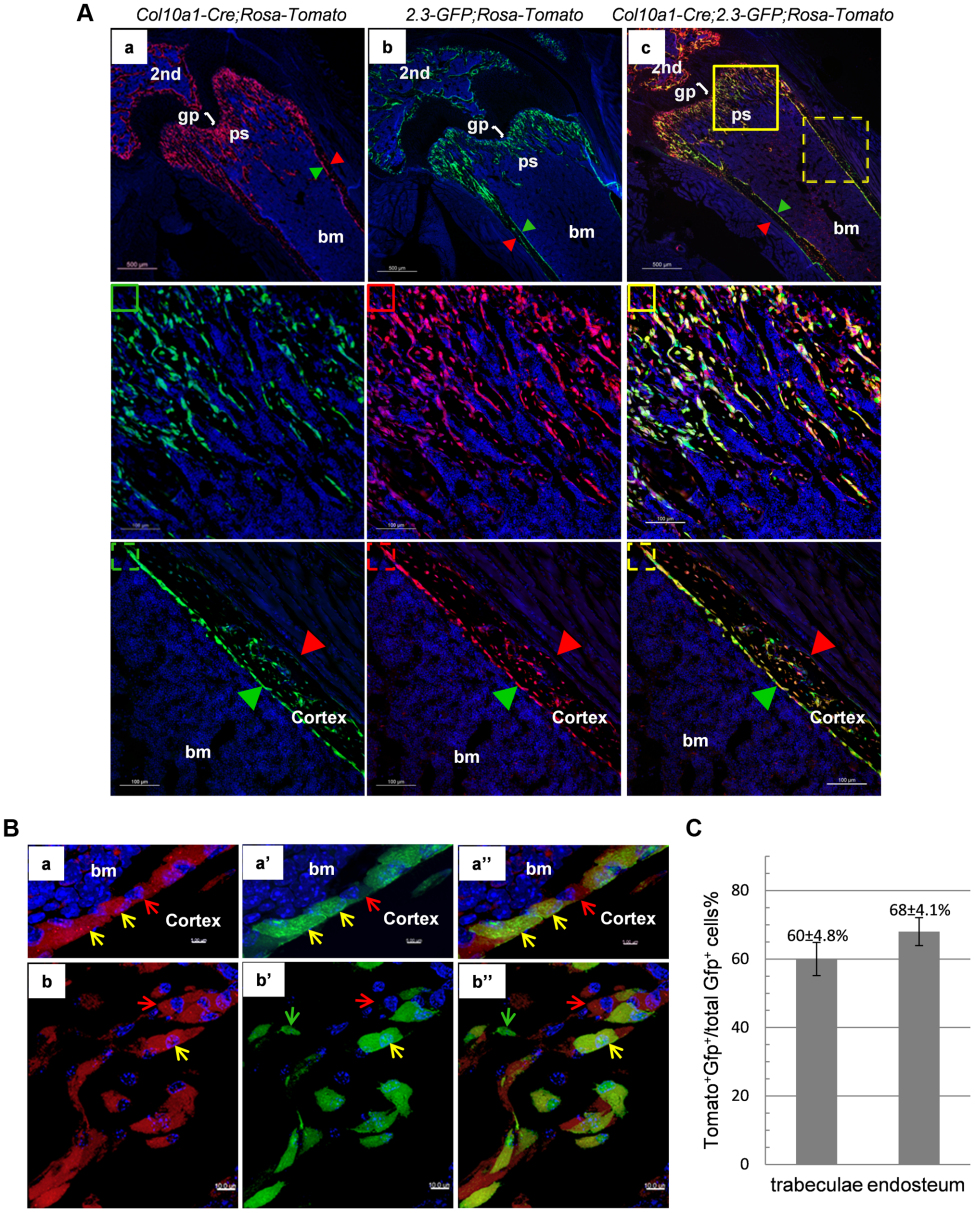
Cellular colocalization of the chondrocyte-derived tomato marker and a 2.3Col1-GFP osteoblast specific marker in femur sections of *Col10a1-Cre;2.3Col1-GFP;ROSA-tdTomato* triple transgenic mice. A: The femur fluorescence images of 3-week-old *Col10a1-Cre;2.3Col1-GFP;ROSA-tdTomato* mouse and *Col10a1-Cre; ROSA-tdTomato* and *2.3Col1-GFP;ROSA-tdTomato* control mice. Panel a: only Tomato^+^ cells (red) no EGFP^+^ (green) cells were present in *Col10a1-Cre; ROSA-tdTomato* control mice. Panel b: only EGFP^+^ cells no Tomato^+^ cells were present in *2.3Col1-GFP; ROSA-tdTomato* control mice. Panel c: Tomato^+^, EGFP^+^ and Tomato^+^EGFP^+^ cells were distributed in the trabecular region, on the endosteum and in the secondary ossification center. The solid square in panel c indicates the enlarged trabeculae region; The dashed square in panel c indicates the enlarged cortical region; The green squares: blue and green channels; The red squares: blue and red channels; The yellow squares: blue, green and red channels; Red arrowhead: periosteum; Green arrowhead: endosteum. 2nd: secondary ossification center. B: Magnified images of cells in A-c. Panels a, a′ and a″: magnified cortical region. a: blue and red channels; a′: blue and green channels; a″: blue, red and green channels. Panels b, b′ and b″: magnified trabecular region. b: blue and red channels; b′: blue and green channels; b″: blue, red and green channels. Red arrows indicate Tomato^+^EGFP^−^ cells. Green arrows indicate Tomato^−^GFP^+^ cells. Yellow arrows indicate Tomato^+^EGFP^+^ cells. Bm: bone marrow. C: The percent quantitation of EGFP^+^ cells which were derived from *Col10a1*-expressing mature chondrocytes in trabecular and cortical regions respectively.

We further generated *Col10a1-Cre; 2.3Col1-GFP; ROSA-tdTomato* triple transgenic mice to assess co-localization of *Col10a1-Cre* induced reporter^+^ cells and an osteoblast-specific marker *in vivo*. In these mice, a Tomato^+^ non-chondrocytic cell represented a *Col10a1-Cre* marked cell whereas a EGFP^+^ cell corresponded to a functional osteoblast [24]. Thus a Tomato and GFP double positive (Tomato^+^EGFP^+^) cell represented an osteoblast derived from *Col10a1*- expressing chondrocytes. In the femur of 3-week-old *Col10a1-Cre;2.3Col1-GFP;ROSA-tdTomato* mice there was an abundance of Tomato^+^EGFP^+^ cells throughout the trabecular and endosteal surfaces (Fig. 5A). These Tomato^+^EGFP^+^ cells in both trabeculae and cortical region were further examined by high resolution microscopy (Fig. 5B). As the Ocn^+^EGFP^+^ cells shown in Fig. 4B, the Tomato^+^EGFP^+^ cells in the femur of 3-week-old *Col10a1-Cre;2.3col1-GFP;ROSA-tdTomato* mice had a typical osteoblast morphology. In the trabecular area, approximately 60% of total EGFP^+^ cells were also Tomato^+^, and around 68% of EGFP^+^ cells on the endosteum surface were positive for Tomato (Fig. 5C and Table S3). This result indicated that the hypertrophic chondrocyte-derived osteoblasts contributed to more than half of the osteoblast population in the 3-week-old mice *in vivo*.

**Figure 6:**
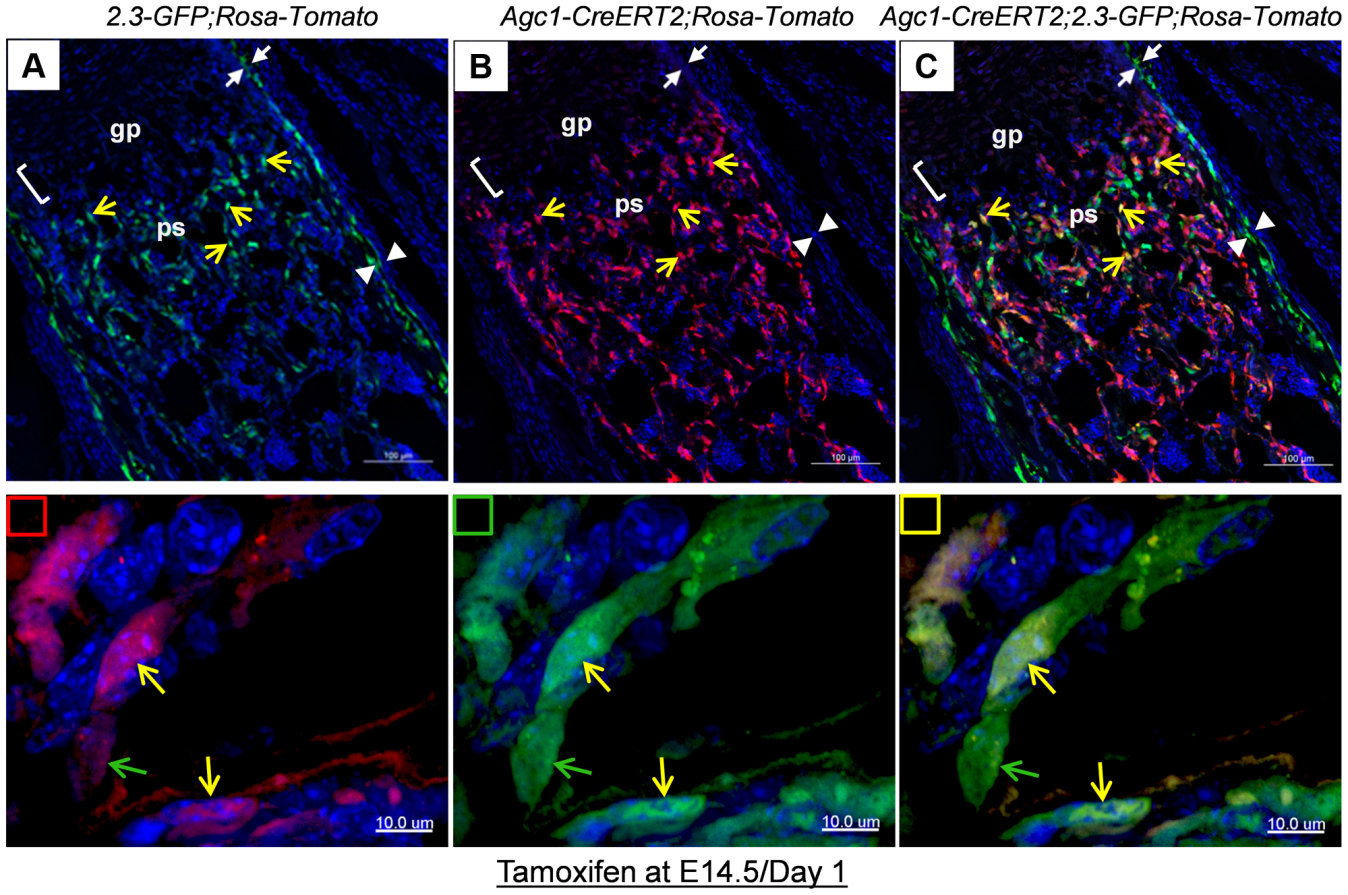
Cellular colocalization of the chondrocyte-derived tomato marker and the osteoblast-specific 2.3Col1-GFP marker in femurs of tamoxifen treated *Agc1-CreERT2;2.3Col1-GFP;ROSA-tdTomato* triple transgenic mice. The femur fluorescence images of postnatal day 1 *Agc1-CreERT2;2.3Col1-GFP;ROSA-tdTomato* mouse treated with tamoxifen at E14.5. Panel a: blue and green channels; Panel b: blue and red channels; Panel c: blue, red and green channels; The lower panels indicated by small squares represent magnified images of cells from top panels. Red square: red and blue channels; Green square: green and blue channels; Yellow square: red, green and blue channels; The green arrows indicate the Tomato^−^EGFP^+^ cells. The yellow arrows indicate the Tomato^+^EGFP^+^ cells. Only EGFP^+^ cells no Tomato^+^ cells were present in the perichondrium (between white arrows) and periosteum (between white arrow heads).

As in the case of *Col10a1-Cre;2.3Col1-GFP;ROSA-tdTomato* mice, Tomato^+^EGFP^+^ cells were also present in the femur of newborn *Agc1-CreERT2;2.3Col1-GFP;ROSA-tdTomato* mice treated with tamoxifen at E14.5 (Fig. 6). The Tomato^+^ and Tomato^+^EGFP^+^ cells were specifically located in the primary spongiosa, but not in perichondrium and periosteum, tissues that were also marked by EGFP expression. This result further substantiated our hypothesis that hypertrophic chondrocytes were capable of differentiating into bone–forming osteoblasts *in vivo*.

Together, these results suggested that osteoblasts, which were derived from chondrocytes in cartilage anlagen constitute a major source of osteoblasts responsible for trabecular and endosteal bone formation in the growing endochondral skeleton.

### Growth plate mature chondrocytes continue to contribute to the osteoblast pool during early postnatal growth

**Figure 7:**
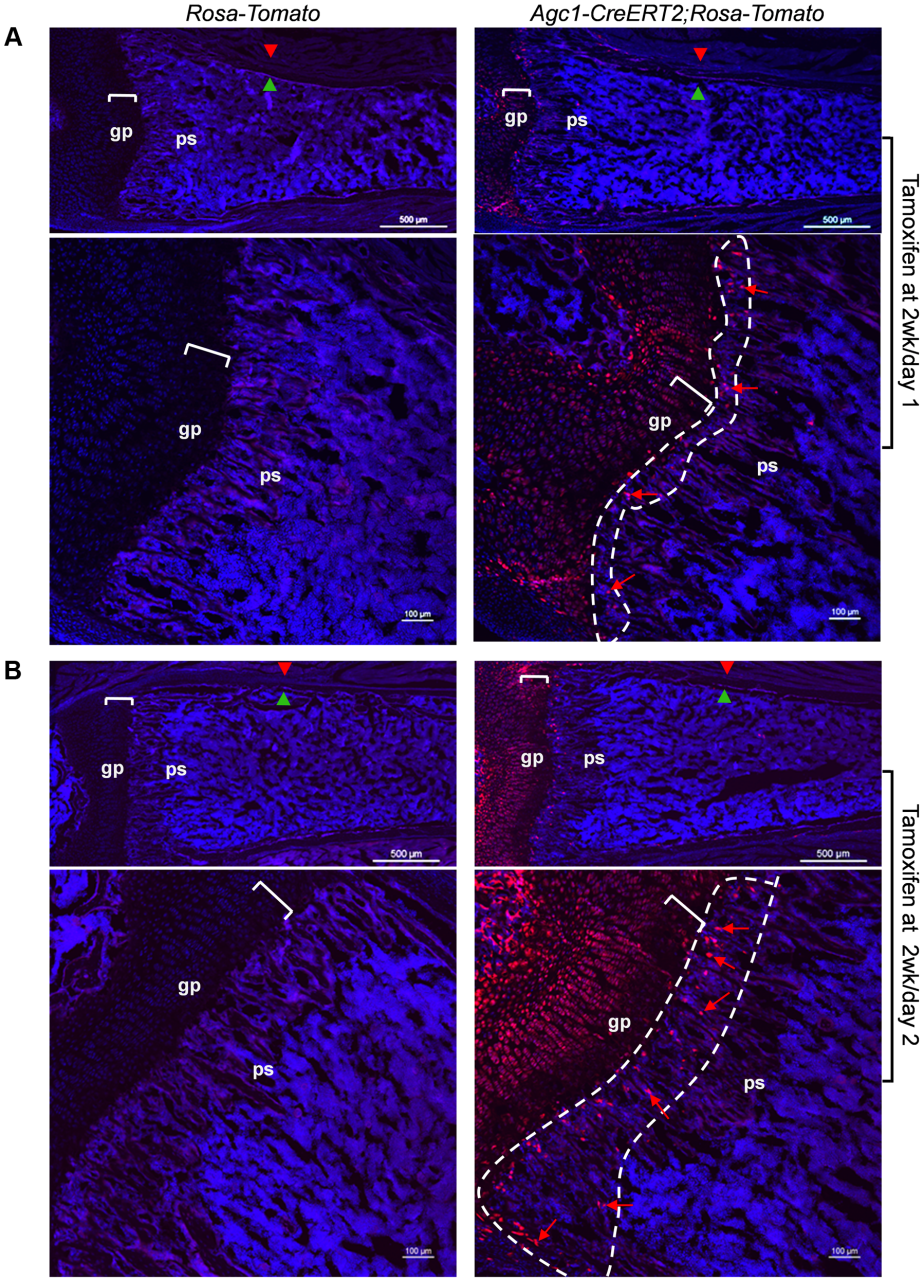
Presence in the primary spongiosa of non-chondrocytic cells derived from postnatal growth plate mature chondrocytes. *Agc1-CreERT2; ROSA-tdTomato* and control mice were treated with tamoxifen at 2 weeks postnatally and were sacrificed at day 1 (A) and day 2 (B) post injection. All images in this figure are images of femur sections. Left panels (A and B): Tomato^+^ cells were completely absent in *ROSA-Tomato* control mice. Right panels (A and B): Tomato^+^ non-chondrocytic cells (red arrows) were present in the primary spongiosa under the growth plate. More Tomato^+^ non-chondrocytic cells were seen in the primary spongiosa in the post injection day 2 mouse, and these Tomato^+^ non-chondrocytic cells of the post injection day 2 mouse were distributed in a wider area than in the day 1 mouse. The white dotted lines outlines the area within which non-chondrocytic Tomato^+^ cells were present. Red arrowhead: periosteum; Green arrowhead: endosteum; gp: growth plate (white brackets); ps: primary spongiosa.

To evaluate whether the growth plate chondrocytes during postnatal growth also contribute to the osteoblast pool in the primary spongiosa as is the case during embryonic development, we generated inducible *Agc1-CreERT2; ROSA-tdTomato* and *Agc1-CreERT2; 2.3Col1-GFP; ROSA-tdTomato* mice to specifically tag and trace the growth plate chondrocytes after birth. At 2 weeks, the *Agc1-CreERT2; ROSA-tdTomato* or *Agc1-CreERT2; 2.3Col1-GFP; ROSA-tdTomato* and control mice were treated with tamoxifen and subsequently sacrificed at different time points after tamoxifen injections. ISH showed that *Agc1* was specifically expressed in growth plate chondrocytes, not in cells in primary spongiosa of 2 and 3-week-old *Agc1-CreERT2; ROSA-tdTomato* and *Agc1-CreERT2; 2.3Col1-GFP; ROSA-tdTomato* mice (Figure S4A). At post tamoxifen day 1, a majority of growth plate chondrocytes were weakly positive for Tomato and very few Tomato^+^ cells were present at the chondro-osseous junction in the primary spongiosa of a *Agc1-CreERT2; ROSA-tdTomato* mouse, while no Tomato^+^ cells were detected in the *ROSA-tdTomato* control (Fig. 7A). At post tamoxifen day 2 in *Agc1-CreERT2; ROSA-tdTomato* mouse, more non-chondrocytic Tomato^+^ cells appeared below the growth plates in the primary spongiosa in addition to the Tomato^+^ chondrocytes. The fluorescence signal of day 2 Tomato^+^ chondrocytes was more intense than the day 1 Tomato^+^ chondrocytes (Fig. 7B). Unlike in the 2-week-old *Col10a1-Cre; Osx ^flox/+^* mice (Fig. 1D), there were no Tomato^+^ cells on the endosteum or embedded within the cortex in 2-week-old *Agc1-CreERT2; ROSA-tdTomato* mice both 1 and 2 days post tamoxifen injection.

**Figure 8:**
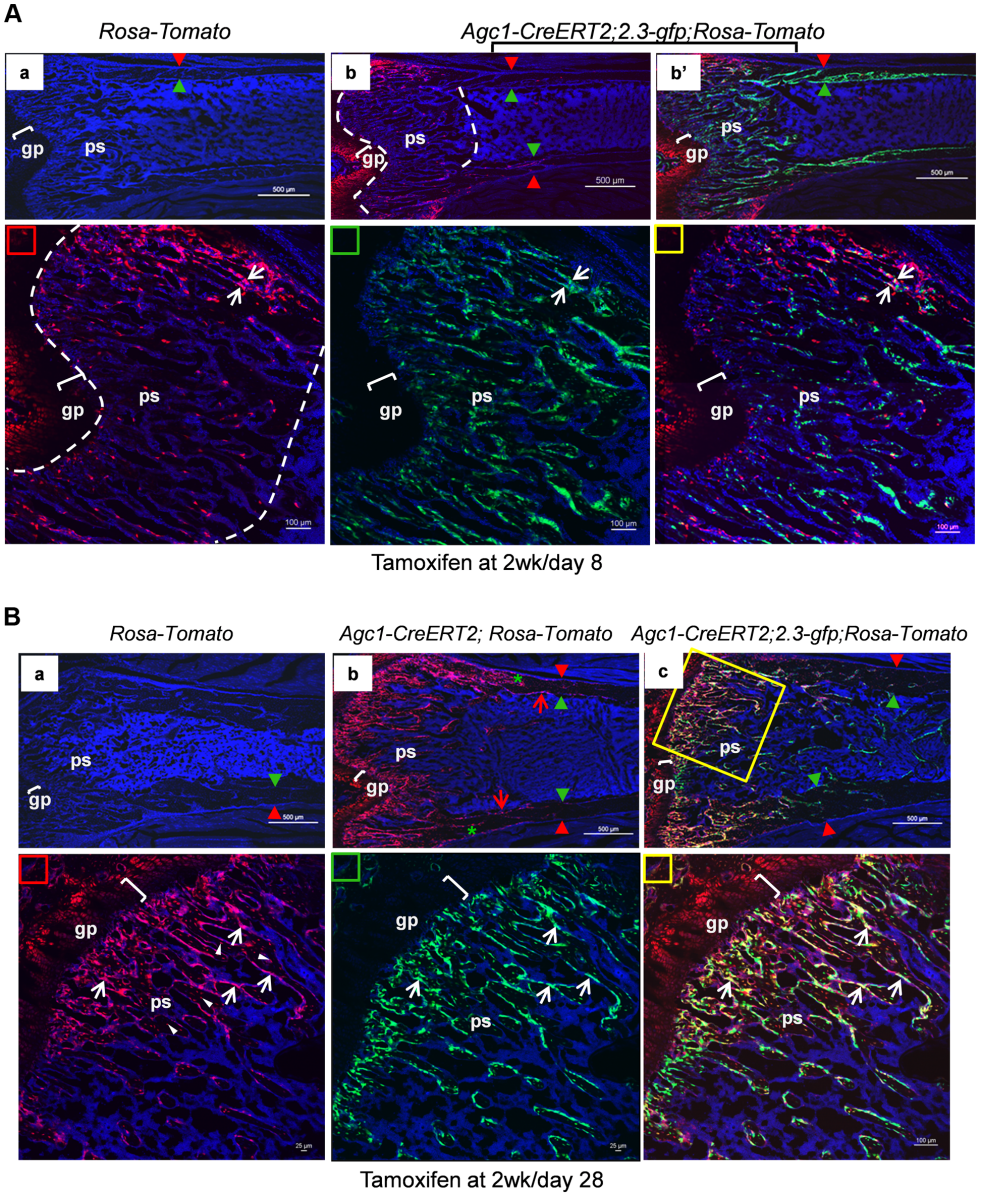
Growth plate mature chondrocytes contribute to the osteoblast pool during postnatal growth. Two-week-old *Agc1-CreERT2; ROSA-tdTomato, Agc1-CreERT2; 2.3-GFP;ROSA-tdTomato* and control mice were treated with tamoxifen and sacrificed 8 days (A) and 28 days post injection (B). All images in this figure are images of femur sections. A: Panel a: neither Tomato nor EGFP signals were detected in the *ROSA-tdTomato* control. Panel b (blue and red channels): the fluorescence image revealed that there were more Tomato^+^ cells in the primary spongiosa in the post injection day 8 *Agc1-CreERT2; 2.3-GFP;ROSA-tdTomato* mouse compared to the post injection day 2 mouse (Fig. 7B right panels), and the Tomato^+^ cells in the day 8 mouse were distributed in an even broader area than in the day 2 mouse. Very few Tomato^+^ cells were found on the endosteum and within the cortex. Panel b′: blue, red and green channels. Lower panels: magnified images of primary spongiosa in b′. Lower left (red rectangle): red and blue channels; Lower middle (green rectangle): green and blue channels; Lower right (yellow rectangle): red, green and blue channels: a few Tomato^+^EGFP^+^ cells (white arrows) were present in the primary spongiosa especially in proximity of the periosteum. B: Panel a: no Tomato or GFP signals were detected in the post tamoxifen day 28 *ROSA-tdTomato* control. Panel b: in the post tamoxifen day 28 *Agc1-CreERT2; ROSA-tdTomato* mouse, not only was the number of Tomato^+^ cells in the primary spongiosa increased compared to the post injection day 8 mouse, but some of these Tomato^+^ cells were on the endosteum (red arrows) and embedded within the cortex (green asterisk). Panel c: there were many more Tomato^+^EGFP^+^ cells (white arrows in magnified lower right panel) in the day 28 *Agc1-CreERT2; ROSA-tdTomato* mouse than in the day 8 mouse (Fig. 8A). Lower left panel: magnified trabecular area of panel c (red and blue channels) showed that there were many Tomato^+^ cells on the trabecular surfaces (white arrows) and embedded within the trabeculae (white arrowheads). Red arrowhead: periosteum; Green arrowhead: endosteum; gp: growth plate (white brackets); ps: primary spongiosa.

At 8 days post tamoxifen treatment, the number of non-chondrocytic Tomato^+^ cells in the primary spongiosa was substantially increased and these cells were distributed in a broader area under the growth plates than in the post tamoxifen day 1 and day 2 mice (Fig. 8Ab). A majority of the non-chondrocytic Tomato^+^ cells in the day 8 primary spongiosa were small in size and as those in day 1 and day 2 mice were not found within the trabeculae or cortex. Only some of the Tomato^+^ cells in the vicinity of periostea displayed a larger and elongated morphology and some of them were also positive for EGFP, suggesting the presence of chondrocyte derived osteoblasts in the primary spongiosa of these mice (Fig. 8Ab′). In the 6-week-old *Agc1-CreERT2; 2.3Col1-GFP; ROSA-tdTomato* mice, which were treated with tamoxifen at 2weeks, the number of non-chondrocytic Tomato^+^ cells was substantially increased and the cells were distributed throughout the breadth of the primary spongiosa. These cells were morphologically similar to those in the vicinity of periostea in the post tamoxifen day 8 mice. A considerable number of Tomato^+^ cells were also embedded within trabeculae and a few were found on the endosteum and within the cortex (Fig. 8B). Moreover, the number of Tomato^+^EGFP^+^ cells were very substantially increased compared to the post tamoxifen day 8 mice. Thus there was a considerable time lag between the initial labeling of growth plate chondrocytes, the presence of abundant chondrocyte-derived non-chondrocytic cells in primary spongiosa and particularly the formation of large numbers of chondrocyte-derived functional osteoblasts. Note that the number of Tomato^+^ cells on the endosteum and within the cortex in these mice was much less than in the 3-week-old *Col10a1-Cre; 2.3Col1-GFP; ROSA-tdTomato* mice (Fig. 5A).

When 11-week-old *Agc1-CreERT2; 2.3Col1-GFP; ROSA-tdTomato* mice were treated with tamoxifen and were sacrificed two weeks later, the Tomato^+^ cells were present in a narrower area under the growth plate (Figure S4B) than in the mice sacrificed 8 days after tamoxifen injection at 2 weeks (Fig. 8A); in these 13-week-old mice there were also very few Tomato^+^EGFP^+^ cells.

Overall, these results indicate that postnatal growth plate chondrocytes continued to contribute to the osteoblasts pool during the period of rapid postnatal growth in juvenile mice.

### Osteoblasts derived from mature chondrocytes in the repair callus are involved in bone fracture healing

Bone fracture healing occurs mostly through processes similar to developmental endochondral ossification, which involves a cartilage intermediate [25], [26]. To test the hypothesis that mature chondrocytes present in the repair callus may be a source of osteoblasts contributing to bone formation during fracture healing, we generated semi-stabilized fractures of the tibia in 2 to 3-month-old *Agc1-CreERT2; ROSA-tdTomato or Agc1-CreERT2;2.3Col1-GFP;ROSA-tdTomato* and control mice and subsequently injected tamoxifen into these mice at 6 to 7 days post-surgery, a time which is prior to or around the time of chondrocyte differentiation [25], [26].

**Figure 9:**
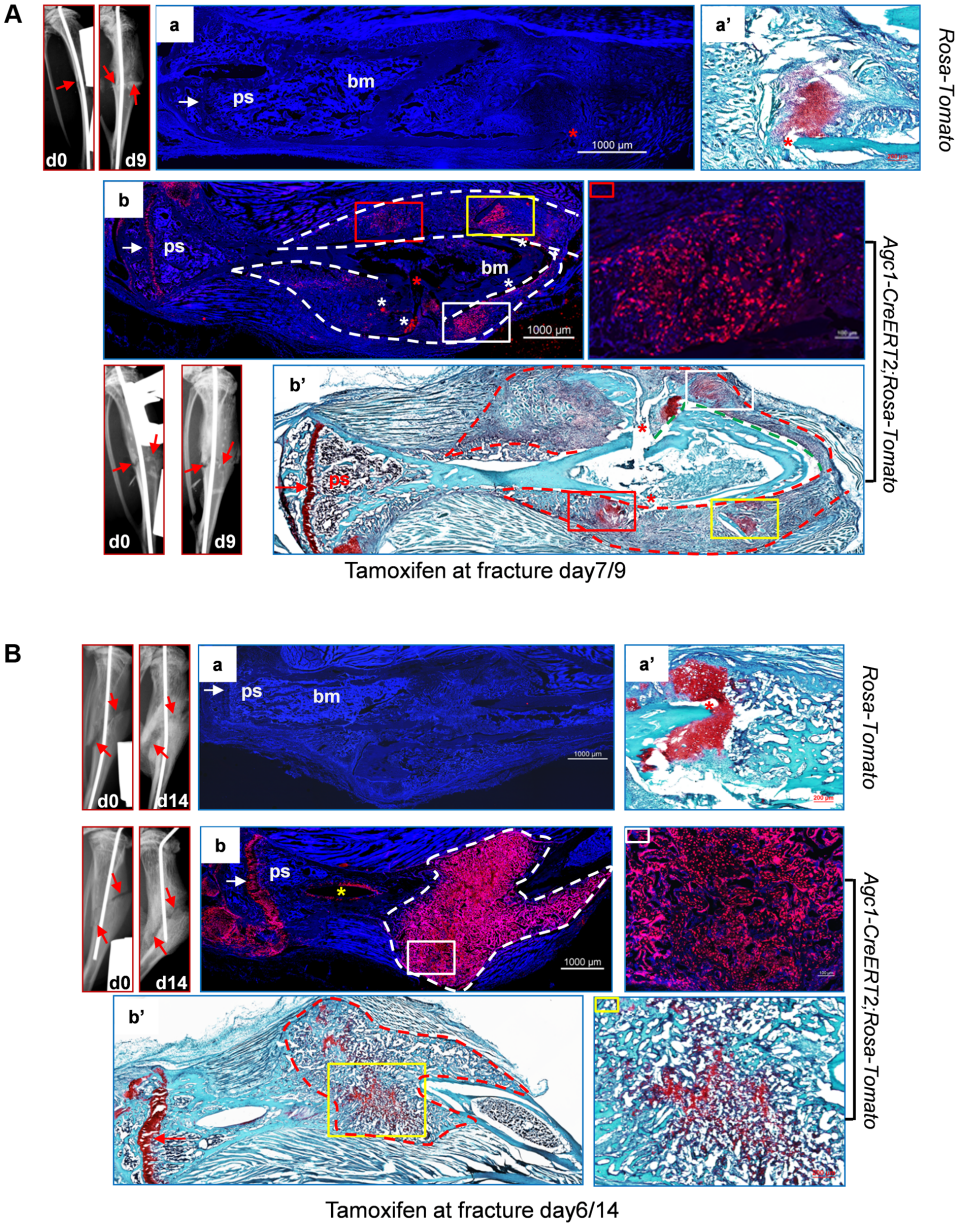
Abundant *Agc1-CreERT2* labeled Tomato^+^ cells were present in the repair callus during fracture healing. A: The left tibia of 2.5-month-old *Agc1-CreERT2; ROSA-tdTomato* mouse and *ROSA-tdTomato* control mouse were subjected to semi-stabilized fracture surgery. At 7 days after surgery, these mice were treated with tamoxifen and were sacrificed 2 days post tamoxifen. The panels left to panel a: X-ray images of fractured tibiae of *ROSA-tdTomato* control mouse. day0: right after surgery; day9: 9 days after surgery. Red arrows indicate the fractures. Panel a: the fluorescence image of fractured tibiae of *ROSA-tdTomato* mouse. Panel a′: Saf-O staining indicates the presence of chondrocytes (red staining) in the repair callus of *ROSA-tdTomato* fractured tibiae. Panel b: fluorescence image of the fractured tibiae of *Agc1-CreERT2; ROSA-tdTomato* mouse, Tomato^+^ cells were present specifically in the growth plate (white arrow) and in the repair callus outlined by the dotted lines. The colored rectangles indicate the locations of Tomato^+^ cells in the callus. The panel right to panel b: magnified fluorescence image of the area marked by a red rectangle in b. White asterisks indicate non-specific fluorescence signals. The white arrow indicates the growth plates. Panel b′: Saf-O staining of the fractured tibiae. The red dotted lines outline the repair callus. The colored rectangles mark the areas of red staining. The matching areas in b and b′ are marked by the same colored rectangles. Red asterisks: fracture sites. The red arrow indicates the growth plate. B: The left tibia of 2-month-old *Agc1-CreERT2; ROSA-tdTomato* mouse and *ROSA-tdTomato* control mouse were subjected to semi-stabilized fracture surgery. At 6 days after surgery, these mice were treated with tamoxifen and were sacrificed 8 days post tamoxifen. Panel a: fluorescence image of fractured tibiae of *ROSA-tdTomato* mouse. Panel a′: Saf-O staining of repair callus in fractured tibiae in panel a. The panels left to panel a: X-ray images of fractured tibiae of *ROSA-tdTomato*. Panel b: fluorescence image of fractured tibiae of *Agc1-CreERT2; ROSA-tdTomato* mouse. Almost all cells in the callus outlined by the white dotted lines were Tomato^+^ cells. The panel right to panel b: magnified fluorescence image of the area marked by the white rectangle. The white arrow indicates the growth plates. Panel b′: Saf-O staining of the fractured tibiae. The red stippled line outlines the repair callus. The image right to b′: the Saf-O staining of area marked by a yellow rectangle in b′. The red arrow indicates the growth plate.

In the tibia of a post-surgery day 9 *Agc1-CreERT2; ROSA-tdTomato* mouse treated with tamoxifen 7 days after surgery, the Tomato^+^ cells were found in the growth plate and in the repair callus but not in the region between these two areas (Fig. 9Ab). No Tomato^+^ chondrocytes were detected in the *ROSA-tdTomato* control (Fig. 9Aa). In these mice 9 days after surgery, chondrocyte differentiation occurred in some but not all of the callus cells. Interestingly, the areas of Tomato^+^ cells in the repair callus were completely matching the chondrocyte areas stained maroon red by Saf-O (Fig. 9Ab′), suggesting that these Tomato^+^ callus cells were in fact chondrocytes in the cartilage callus.

In the tibia of a post-surgery day 14 *Agc1-CreERT2; ROSA-tdTomato* mouse treated with tamoxifen 6 days after surgery, Saf-O staining indicated that the repair callus was partially ossified, showing a mixture of both bone (green) and cartilage (red) tissues (Fig. 9Bb′). However, almost all cells in the repair callus, both in the cartilage and in the bone regions were positive for Tomato (Fig. 9Bb), suggesting the presence of non-chondrocytic Tomato^+^ cells in the repair callus. As in the case of the post-surgery day 9 fractured tibia (Fig. 9Ab), the Tomato^+^ cells were not found in the region between the growth plate and the repair calluses, implying that the Tomato^+^ cells in the repair callus were in fact either callus chondrocytes or cells derived from callus chondrocytes, and hence that these Tomato^+^ cells were not derived from growth plate chondrocytes.

**Figure 10:**
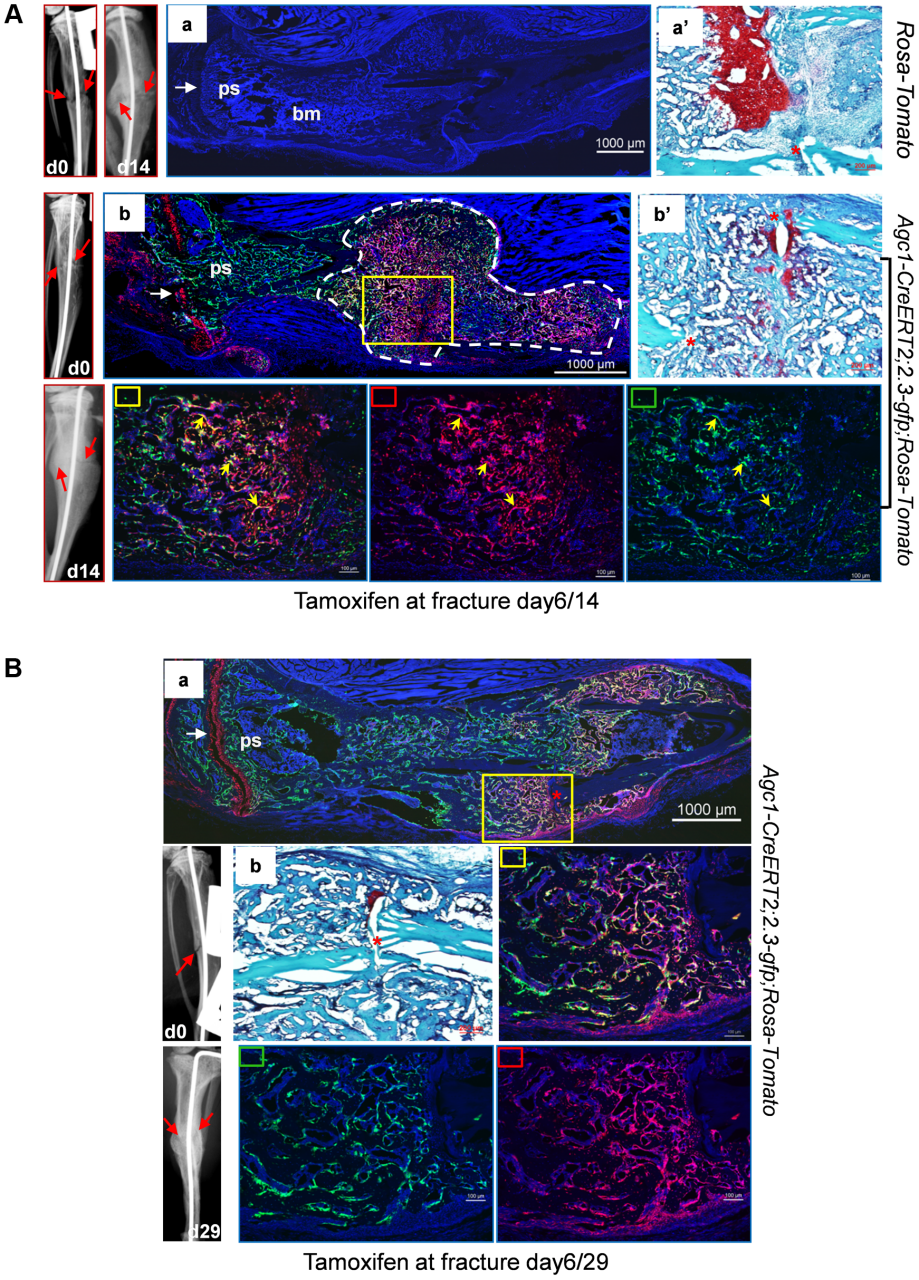
Osteoblasts derived from mature chondrocytes in the repair callus are involved in bone fracture healing. A: Left tibia of a 2.5-month-old *Agc1-CreERT2; 2.3-GFP;ROSA-tdTomato* mouse and a *ROSA-tdTomato* control mouse were subjected to semi-stabilized fracture surgery. At 6 days after surgery, these mice were treated with tamoxifen and were scarified 8 days post tamoxifen. Panel a: fluorescence image shows no Tomato and GFP signals in the fractured tibiae of *ROSA-tdTomato* control. Panel a′: Saf-O staining of the repair callus in a. Images left to a: X-ray images of fractured tibia of control mouse. Panel b: in the fractured tibiae of *Agc1-CreERT2; 2.3-GFP;ROSA-tdTomato* mouse, Tomato^+^ cells are present specifically in growth plate (white arrow) and in the repair callus outlined by dotted white lines. Some of the Tomato^+^ cells were also positive for GFP (Tomato^+^EGFP^+^ indicated by yellow arrows). The white arrow indicates the growth plate, which was partly broken by the pin inserted into the fractured tibia. Panel b′: Saf-O staining of the callus in b. B: The left tibia of 2.5-month-old *Agc1-CreERT2; 2.3-GFP;ROSA-tdTomato* mouse was subjected to semi-stabilized fracture surgeries. At 6 days after surgery, the mouse was treated with tamoxifen and scarified 23 days post tamoxifen. Panel a: the fluorescence image revealed that there were more Tomato^+^EGFP^+^ cells in the callus compared to the callus collected 14 days after surgery (Fig. 10A). Rectangle marked panels: magnified images corresponding to the yellow rectangle in panel a. Middle right panel: blue, red and green channels; Bottom middle panel: blue and green channels; Bottom right panel: blue and red channels. Panel b: Saf-O staining shows very little red tissue, suggesting that the callus was almost all ossified.

In the repair callus of a post-surgery day 14 *Agc1-CreERT2; 2.3Col1-GFP; ROSA-tdTomato* mouse treated with tamoxifen at 6 days after surgery, many of the Tomato^+^ cells were also positive for GFP (Tomato^+^EGFP^+^), implying that the mature chondrocytes present in the repair callus have the ability to become *Col1a1*-expressing bone forming osteoblasts (Fig. 10Ab, Figure S5a).

At day 29 post-surgery, ossification was almost complete in the repair callus of an *Agc1-CreERT2; 2.3Col1-GFP; ROSA-tdTomato* mouse treated with Tamoxifen 6 days post-surgery (Figure S5b). The number of Tomato^+^EGFP^+^ cells was very substantially increased compared to the day 14 callus (Fig. 10Ba).

Thus the appearance of Tomato^+^ cells in the early callus, their localization corresponding to the cartilage callus, the absence of Tomato^+^ cells between the growth plate and the callus, and the presence of abundant Tomato^+^EGFP^+^ cells in the ossifying callus strongly suggested that chondrocytes in the repair callus were a source of osteoblasts involved in bone fracture healing.

## Discussion

Our objective was to follow the fate of chondrocytes in endochondral ossification and to test the hypothesis whether chondrocytes may undergo transdifferentiation into functional osteoblasts *in vivo*.

In studying the fate of labeled chondrocytes in both *Col10a1-Cre*-containing embryos and tamoxifen treated *Agc1-CreERT2*–containing embryos, we observed that, in addition to labeled chondrocytes and hypertrophic chondrocytes, from E15.5 on numerous labeled cells with a different morphology than that of hypertrophic chondrocytes were present in the primary ossification center, where neither *Col10a1-Cre* nor *Agc1-CreERT2* were expressed. Later labeled cells were found within the matrix of bone trabeculae and postnatally in the endosteum and within the cortical bone. The reporter^+^ cells were lining the endosteum continuously from the distal growth plate down to the femur shaft. The area of the endosteum covered by reporter^+^ cells was gradually increasing with time, from up to mid-shaft at 2weeks to about three quarts of the length at 1 month after birth. Although we occasionally observed very rare reporter^+^ cells in the perichondrium or periosteum area, the number of these reporter^+^ cells were too low to account for the abundant reporter^+^ cells in the primary spongiosa.

In *Col10a1-Cre;reporter* embryos the appearance of the non-chondrocytic labeled cells in the primary spongiosa occurred synchronously with the onset of primary ossification. However, the use of *Agc1-CreERT2;reporter* embryos allowed us to control the timing of Cre recombination in chondrocytes and the subsequent appearance of labeled cells in the primary ossification centers by varying the timing of tamoxifen administration.

The tamoxifen pulse experiments with *Agc1-CreERT2; ROSA26R* embryos (Fig. 3, Figure S2B, Figure S2C and Table S1) clearly established that the labeling of chondrocytes and the appearance of non-chondrocytic cells in the primary spongiosa were two sequential events and that the latter could be dissociated from the start of osteogenesis. These experiments provided compelling evidence that the labeled cells in the primary ossification centers were derived from chondrocytes, and that their presence was not due to a hypothetical Cre activity in emerging osteoblasts.

The finding that the labeled cells present in the trabecular and endosteal regions of *Col10a1-Cre*-containing mice expressed two osteoblast markers namely Osteocalcin and a osteoblast-specific *Col1a1* promoter-driven EGFP strongly suggested that these cells were functional osteoblasts. In the femur trabeculae of tamoxifen-treated *Agc1-CreERT2*-containing mice the presence of chondrocyte-derived osteoblasts was also confirmed by co-expression of the tomato reporter and EGFP driven by the osteoblast-specific *Col1a1* promoter. Our results further revealed that about 60 percent of osteoblasts in the femurs of 3- and 4-week-old mice were derived from chondrocytes. These numbers were computed in *Col10a1-Cre;reporter* mice, although one cannot completely exclude that the 60 percent figure maybe an overestimate, if for the instance there was the unlikely possibility of an ectopic *Col10a1-Cre* promoter activity. The labeling of chondrocytes after a single tamoxifen injection in pregnant females of *AgcCreERT2* embryos was unlikely to be complete. In the newborn *Agc1-CreERT2; 2.3Col1-GFP; ROSA-tdTomato* mice treated with tamoxifen at E14.5 (Fig. 6), and in the 3-week and 6-week-old mice treated with tamoxifen at 2-week (Fig. 8), some of the osteoblasts (EGFP^+^ cells) in the primary spongiosa were derived from mature chondrocytes prior to tamoxifen injections. This population of EGFP^+^ cells would therefore not be labeled by *Agc1-CreERT2* and these cells would thus be negative for Tomato. Hence, the number of Tomato^+^ EGFP^+^ cells would not reflect the actual number of chondrocyte-derived osteoblasts present in these mice.

Our data revealed that both mature chondrocytes in cartilage primordia prior to the establishment of growth plates, and mature chondrocytes in established growth plates, transdifferentiated and contributed to the osteoblast pools. The reporter^+^ cells labeled by *Agc-CreERT2* around E14.5, before the establishment of the growth plate persisted into the growth period after birth, since they were still found on the surfaces of trabeculae and endosteum, and embedded within the bone matrix at 2 weeks and 1 month (Figs. 3B and 3C). After the establishment of growth plates, mature chondrocytes in growth plates maintained their ability to become osteoblasts both during late embryonic development and postnatal growth (Figure S2D, Figs. 7 and 8). During the rapid growth period at 2 weeks, the *Agc1-CreERT2* induced more non-chondrocytic reporter^+^ cells in the primary spongiosa in less time (Figs. 7 and 8) than during the late growth period at 11 weeks (Figure S4B). These results suggested the hypothesis that the contribution of growth plate chondrocytes to the osteoblast pool could possibly be associated with growth plate activity and that this contribution might even eventually stop sometime after the growth period.

After the growth period the *Col10a1-Cre* induced reporter^+^ cells were still present in the metaphyseal and cortical regions in 6-month-old mice (Figure S1F). However, more of these cells were embedded within the bone matrix, and less of them were found on the bone surfaces, compared to the 2- and 3-week-old mice (Fig. 1D and Fig. 5A), implying that the number of active osteoblasts derived from chondrocytes was likely reduced after the growth period. At 8-month, there were almost no *Col10a1-Cre* induced reporter^+^ cells in the primary spongiosa and very few were on the bone surfaces. In addition, the fluorescence intensities of Tomato^+^ cells were much weaker than that in the younger mice (Figure S1G). These results implied that the *Col10a1-Cre* induced cells in the 8-month-old mice were probably derived from the growth plate chondrocytes during the growing period, further suggesting an association between the appearance of chondrocyte-derived osteoblasts and the growth plate activities.

In the fractured tibiae, *Agc1-CreERT2* induced Tomato^+^ cells were present specifically in the articular cartilage, growth plate and repair calluses when tamoxifen was injected at post surgery day 6 or day 7 around the time of chondrocyte differentiation in the callus [25], [26]. The *Agc1-CreERT2* induced Tomato^+^ cells continued to be present in calluses after 14 and 29 days post-surgery, when *Agc1-CreERT2* was no longer active, suggesting that these Tomato^+^ cells were either callus chondrocytes labeled at the time of tamoxifen injection around post fracture day 7 or cells derived from these labeled chondrocytes. Importantly there were no significant numbers of Tomato^+^ cells observed in the region between growth plates and calluses during the repair process, indicating that the Tomato^+^ cells present in calluses were unlikely derived from growth plate chondrocytes (Figs. 9 and 10). The Tomato^+^EGFP^+^ cells were found in the partially ossified callus at post surgery day 14, and the appearance of these Tomato^+^EGFP^+^ cells was temporally and spatially associated with callus ossification. These data substantiated that chondrocytes in the repair callus were a clear source of osteoblasts responsible for bone formation during fracture healing.

Several laboratories have attempted to address the question whether chondrocytes had the ability to transdifferentiate into osteoblasts. In an earlier *ex vivo* experiment, in which vascular invasion and endochondral ossification were inhibited by removal of the perichondrium, *Col1a1* expressing cells were observed in an area between the cartilage anlagens [12]. Although these cells were not further characterized, they were hypothesized to be either hypertrophic chondrocytes arrested at a terminal stage of differentiation or having undergone transdifferentiation. The drawback of this *ex vivo* experiment was the absence of perichondrium, which may be essential for complete chondrocytes transdifferentiation. We consider the present study as an extension of this previous experiment.

In an *in vivo* study in *Col2a1-CreER; ROSA26R* embryos specific cells at the cartilage/perichondrium interface, which expressed the *Col2a1-CreER* transgene, were shown to give rise to some likely osteoblasts mainly in the endosteal region [3]. While this study concluded that hypertrophic chondrocytes did not make a detectable contribution to the osteoblast/osteocyte pool in the central metaphyseal regions under the growth plate within a 3-day time frame, this may be explained by the short time span after tamoxifen administration. In another *in vivo* study transgenic mice were used harboring both a *Col10a1-mCherry* and either a *2.3kbCol1a1-Emerald* transgene or a *3.6kbCol1a1-Topaz* osteoblast-specific transgene. MCherry fluorescence was detected in the primary spongiosa but not *mCherry* mRNA. Although much of the mCherry fluorescence was attributed to decaying hypertrophic chondrocytes some of it was present in live cells which did not express the emerald or topaze transgenes. It was speculated that these cells might be a rare population of hypertrophic chondrocytes showing a phenotype different from or preceding that of differentiated osteoblasts. However, the study concluded that transdifferentiation was unlikely to be involved in this phenotype. [13]. Also here, the time lag between labeling of chondrocytes and their appearance as osteoblast marker-expressing cells, as observed in our work, may explain the seeming discrepancy between the studies.

**Figure 11:**
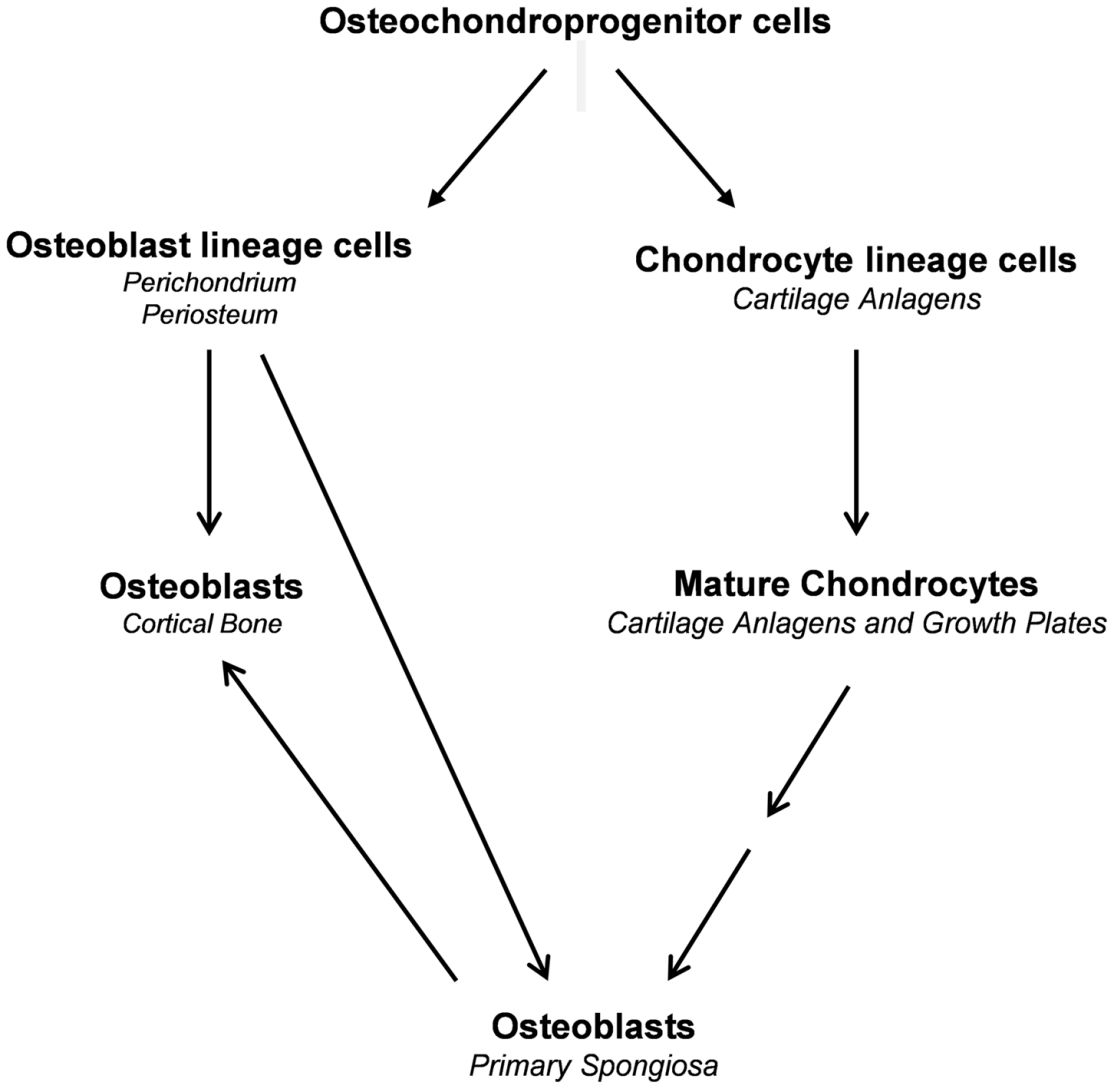
Proposed model illustrating the sources of osteoblasts in endochondral bone.

During endochondral bone formation, osteoblast lineage cells first segregate from Sox9-expressing mesenchymal progenitor cells in a process that involves canonical Wnt/β-catenin signaling as well as *Runx2* and *Osx* expression and silencing of *Sox9* expression and Sox9 activity to form the perichondrium and periosteum [16], [27], [28], [29], [30]. These osteoblast precursors differentiate further into osteoblasts to form the initial cortical bone. They also invade the cartilage anlagen together with osteoclasts and blood vessels to differentiate into mature osteoblasts that generate bone trabeculae [3]. In parallel to the segregation of osteoblast lineage cells, chondrogenic cells segregate from the same progenitors and in a stepwise fashion differentiate into chondrocytes, which express high levels of Sox9 to form the initial cartilage anlagen [31]. The last step in this chondrogenic pathway is the formation of hypertrophic chondrocytes, in which *Sox9* is no longer expressed. Our current results provide evidence that hypertrophic chondrocytes both before and after the establishment of the growth plate constitute a second major source of osteoblasts that participate in the formation of trabecular and cortical bones (Fig. 11). This property of these chondrocytes to be a direct source of osteoblasts persists until at least three months of age. The same property of chondrocytes to give rise to osteoblasts is also very active in the bone fracture repair process.

Recent advances have reshaped the conventional view of differentiated mammalian cells in terms of their plasticity [32]. As is the case in other instances of transdifferentiation [33] it is possible that hypertrophic chondrocytes might first dedifferentiate, then proliferate before these cells redifferentiate into osteoblasts. Our preliminary unpublished experiments revealed the existence of cells in the bone marrow that were derived from *Col10a1*-expressing chondrocytes. These cells displayed properties of mesenchymal progenitor cells and were able to differentiate into osteoblasts, chondrocytes and adipocytes *in vitro*, although we do not know whether these cells had the ability to become osteoblast cells *in vivo*. This preliminary result suggests the possibility that “hypertrophic chondrocytes to osteoblasts” transdifferentiation may indeed involve a dedifferentiation and then a redifferentiation process. We noted that in juvenile mice the chondrocyte-derived reporter^+^ cells, which were negative for osteoblast markers, persisted in the primary spongiosa for a considerable amount of time before becoming functional osteoblasts (Figs. 8A and 8B). We further speculate that these two hypothetical steps may be independently regulated by different signaling pathways.

Collectively, our mouse models provide evidences that chondrocytes, both in cartilage anlagen and in growth plate, are a direct source of osteoblasts responsible for endochondral bone formation during development and postnatal growth. Likewise, chondrocytes in repair calluses undergo transdifferentiation to become osteoblasts contributing to callus ossification in fracture repair. We speculate that chondrocyte-derived osteoblasts may also be involved in other endochondral ossification processes, such as osteophyte formation in osteoarthritis [34] and heterotopic ossification in fibrodysplasia ossificans progressiva disorder [35], [36].

While this manuscript was in the final stage of revision, a study [37] was published which reached similar conclusions as those in the present paper.

## Methods

### Ethics statement

All mice work were conducted strictly according to the NIH guidelines.

### Generation of embryos and mice for analysis

The *Col10a1-Cre* mice were generated by Dr. Klaus von der Mark [18]. The *Agc1-CreERT2* and *Osx ^flox/flox^* mice were previously generated in Dr. de Crombrugghe's laboratory [19], [23]. The *2.3Col1a1-GFP* mice were generously provided by Dr. David Rowe [24]. The *ROSA26R* (B6.129S4-*Gt(ROSA)26Sor^tm1Sor^*/J, stock number: 003474) and *ROSA-tdTomato* (B6;129S6-*Gt(ROSA)26Sor^tm9(CAG-tdTomato)Hze^*/J, stock number:009705) mice were purchased from the Jackson Laboratory. The mice were injected intraperitoneally with1.5 mg/10 g body weight of tamoxifen solution on the desired embryonic days. Tamoxifen was dissolved in 10% ethanol and 90% corn oil. Tamoxifen (Sigma, T5648) was first mixed with 1/10^th^ volume of ethanol and then emulsified in corn oil (Sigma,C8267).

### X-Gal staining of β-galactosidase activity

The embryos or mice were fixed in 0.2% glutaraldehyde and 0.8% formaldehyde (pH 7.5 for embryos, pH 7.8 for mice) at room temperature for 1 hour and stained in 1 mg/ml X-gal overnight at room temperature. After staining, the samples were post-fixed in 4% paraformaldehyde in PBS (pH 7.4) at 4°C overnight and dehydrated for paraffin embedding. The post-fixed bones were decalcified prior to embedding.

### *In situ* hybridization

ISH with digoxin (DIG)-labeled riboprobes was carried out on 10-µM frozen sections as previously described [38]. Instead of dehydration for paraffin sections, the frozen sections were washed twice in PBS (pH 7.4) for 5 minutes.

### Immunohistochemistry and immunofluorescence

Bones fixed in 4% paraformaldehyde in PBS (pH 7.4) overnight were decalcified and embedded for CryoJane frozen sections as previously described [39]. Sections were treated with hyaluronidase (Sigma, H4272, 2 mg/ml in PBS [pH 5.0]) at 37°C for 20 minutes for embryonic samples or 30 minutes for postnatal samples and incubated with anti-EGFP (Invitrogen A11122) at room temperature for 1 hour or with anti-mouse collagen type I (Millipore AB765P) at 4°C overnight. For IHC, the sections were then incubated with HRP polymer conjugates (Invitrogen 87-9263) and developed with a Vectastain ABC kit (Vector PK-4000). For IF, the sections were then incubated with anti-rabbit Alexa Fluor 555 (Invitrogen A21428). For anti-EGFP and anti-col1a1 double IF, after incubation with anti-EGFP and anti-rabbit Alexa Fluor 555, the sections were incubated with Alexa Fluor 488-labeled (Invitrogen A10468) anti-mouse collagen type I at 4°C overnight. For anti-EGFP and anti-Ocn double IF, after incubation with anti-EGFP (abcam ab13970) and anti-Ocn (A93876), the sections were incubated with anti-chicken Alexa 488 (abcam) and anti-rabbit Alexa Fluor 555 (Invitrogen A21428). All IF sections were mounted with Prolong Gold antifade reagent with DAPI (Invitrogen P36931).

### Semi-stabilized tibia fracture healing models

The 2 to 3-month-old *Agc1-CreERT2; ROSA-tdTomato* or *Agc1-CreERT2; 2.3Col1-GFP; ROSA-tdTomato* and control mice were used in the fracture surgery. The procedure was described in [3].

### Confocal microscope and analysis

Fluorescence cell images were captured using a A1 Laser scanning confocal microscope made by Nikon Instruments.

## Supporting Information

Figure S1 A: Illustration of *Osx* floxed allele and recombined *Osx* floxed allele. B: Panels a and b: LacZ staining of E15.5 *Col10a1-Cre; ROSA26R* hind limbs showed that *Col10a1-Cre* was active in hypertrophic chondrocytes, not in perichondrium (red arrows) or periosteum (green arrows). Panel a: femur; b: tibia. hp: hypertrophic chondrocytes; poc: primary ossification center. C: ISH revealed that there were practically no *Col10a1*-expressing cells in the primary spongiosa of the femurs of E16.5 *and* E18.5 *Osx^flox/+^* embryos and there were no *Cre*-expressing cells in the primary spongiosa of the femur of a E18.5 *Osx^flox/+^* embryo. Black brackets indicate hypertrophic zones; ps: primary spongiosa. D: Anti-EGFP IF showed that no EGFP^+^ cells were observed in the femur of a 2-week-old *Osx ^flox/+^* control mouse. Green arrowhead: endosteum; Red arrowhead: periosteum. E: Anti-EGFP IF showed that no EGFP^+^ cells were observed in the calvariae of E 18.5 *Col10a1-Cre; Osx^flox/+^* embryo, while abundant EGFP^+^ cells were present in the basisphenoid bone of the same section. Panels a and a′: calvariae indicated by red arrows; b and b′: basisphenoid bone indicated by white arrows. Panels a and b: anti-EGFP (red); a′ and b′: anti-EGFP and DAPI (blue). F: The femur fluorescence images of 6-month-old *Col10a1-Cre; ROSA-tdTomato;Osx^flox/+^* mice and *ROSA-tdTomato;Osx^flox/+^* control mice. Red arrowhead: periosteum; Green arrowhead: endosteum; gp: growth plate (white brackets); ps: primary spongiosa; bm: bone marrow. G: The femur fluorescence images of 8-month-old *Col10a1-Cre; ROSA-tdTomato* (b and b′) and *ROSA-tdTomato* control mice (a). The white arrow indicates the bright Tomato^+^ cells in the growth plate of *Col10a1-Cre; ROSA-tdTomato* mouse. Red arrowhead: periosteum; Green arrowhead: endosteum; gp: growth plate (white brackets); ps: primary spongiosa; bm: bone marrow.
(PDF)

Figure S2 A: LacZ staining of a E14.5 *Agc1-CreERT2; ROSA26R* embryos treated with tamoxifen at E13.5. Panels a, a′: E14.5 humerus; b, b′: tibia and fibula. The data show that *Agc1-CreERT2* mediated recombination took place specifically in chondrocytes, not in perichondrium (red arrows). B: LacZ staining of a E13.5 *Agc1-CreERT2; ROSA26R* embryo treated with tamoxifen at E11. C: LacZ stained femur sections of *Agc1-CreERT2; ROSA26R* embryos, which were collected 9, 18 and 24 hours (E16.5) after tamoxifen injection at E15.5. Black brackets indicate hypertrophic zone and the black arrows designate the non-chondrocytic LacZ^+^ cells in primary spongiosa. D: LacZ stained femur (left panels) and tibia (right panels) sections of postnatal day 2 *Agc1-CreERT2; ROSA26R* mouse, born to a pregnant female treated with tamoxifen at E17.5. Primary ossification in femurs occurs earlier than in tibiae. The LacZ ^+^ cells in the primary spongiosa of femur (left panels) were likely derived from mature chondrocytes of completely established growth plates, whereas the LacZ^+^ cells in the primary spongiosa of tibiae (right panels) were likely derived from mature chondrocytes prior to growth plate formation. E: IF with anti-EGFP indicated that no EGFP^+^ cells were observed in the calvariae of 2-week-old *Agc1-CreERT2; Osx^flox/+^* mouse, an offspring of female treated with tamoxifen at E14.5, while abundant EGFP^+^ cells were present in the basisphenoid of the same section. Panels a and a′: calvariae between the red arrows; b and b′: basisphenoid designated by white arrows. Panels a and b: anti-EGFP (red); a′ and b′: anti-EGFP and DAPI (blue). gp: growth plate (white brackets).
(PDF)

Figure S3 A: Double IF with anti-EGFP and anti-Col1a1 showed that EGFP^+^ (*Osx^−/+^*) cells (white arrows) were associated with Col1a1 in the femurs of E18.5 *Col10a1-Cre; Osx^flox/+^* embryos. a: DAPI (blue) and EGFP (red); b: EGFP and Col1a1 (green); c & c′: DAPI, EGFP and Col1a1. White brackets: growth plate (gp); ps: primary spongiosa; bm: bone marrow. B: IHC with anti-BSP reveals that many of the LacZ^+^ cells (red arrows) in the primary spongiosa of the femur of a E16.5 *Agc1-CreERT2; ROSA26R* embryo were directly surrounded by BSP positive bone matrix (brown). hy: hypertrophic zone (red bracket).
(PDF)

Figure S4 A: *Agc1* ISH of femurs of 2-week-old *Agc1-CreERT2;ROSA-tdTomato* (left) and 3-week-old *Agc1-CreERT2; 2.3-GFP;ROSA-tdTomato* (right) mice. B: The fluorescence images of femur sections of 13-week-old *2.3-GFP;ROSA-tdTomato* (a) and *Agc1-CreERT2;2.3-GFP;ROSA-tdTomato* (b, b′ and b″).mice, which were treated with tamoxifen at 11 weeks. The Tomato^+^ cell in the primary spongiosa were distributed in the area outlined by the white dotted lines.
(PDF)

Figure S5 The images of Saf-O stained fractured tibia of 2.5-month-old *Agc1-CreERT2;2.3-GFP;ROSA-tdTomato*. Panel a: the mouse was injected with tamoxifen at 6 days after fracture surgery and was sacrificed 8 days after tamoxifen treatment. Panel b: the mouse was injected with tamoxifen at 6 days after fracture surgery and was sacrificed 23 days after tamoxifen treatment. Red arrow designates growth plate.
(PDF)

Table S1 Dynamics of CreERT2 inducibility by tamoxifen during the skeletal development of *Agc1-CreERT2;ROSA26R* embryos.
(PDF)

Table S2 Estimation of the percent of mature osteoblasts (Ocn^+^) that are derived from chondrocytes (EGFP^+^Ocn^+^) in the trabecular and endosteal regions of 1-month-old *Col10a1-Cre;Osx^flox/+^* mice. The 20× images of anti-Ocn and anti-GFP stained femur sections were used for counting. For the trabecular region, the total numbers of EGFP^+^Ocn^+^ (or Ocn^+^) cells for each sample were the sums of EGFP^+^Ocn^+^ (or Ocn^+^) cells from 4 overlapping images covering the trabecuar region (n = 4). For the endosteal region, the total numbers of EGFP^+^Ocn^+^ (or Ocn^+^) cells for each sample were the sums of EGFP^+^Ocn^+^ (or Ocn^+^) cells from 4 overlapping cortical images (n = 3). NIS-Elements AR software program was used for counting.
(PDF)

Table S3 Estimation of the percentage of osteoblasts (EGFP^+^) that are derived from chondrocytes (Tomato^+^EGFP^+^) in the trabecular and endosteal regions of 3-week-old *Col10a1-Cre;2.3Col1-GFP;ROSA-tdTomato* triple transgenic mice. The numbers of EGFP^+^ cells and Tomato^+^EGFP^+^ cells were obtained using the same method described in Table S2 (n = 5).
(PDF)
