# Supplementary figures and images for: Chondrocytes Transdifferentiate into Osteoblasts in Endochondral Bone during Development, Postnatal Growth and Fracture Healing in Mice
Source: PLoS Genet. 2014 Dec 4;10(12):e1004820. doi: 10.1371/journal.pgen.1004820 (PMC4256265; doi:10.1371/journal.pgen.1004820)

**A**

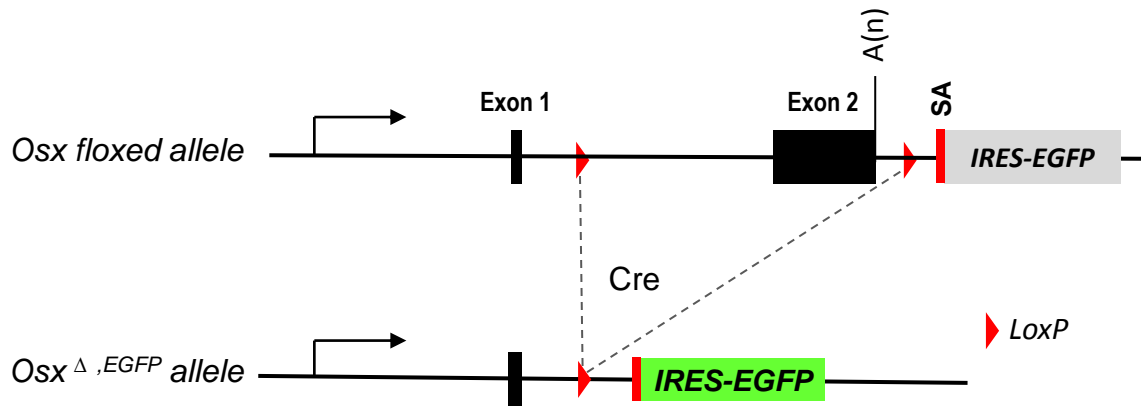

**B**

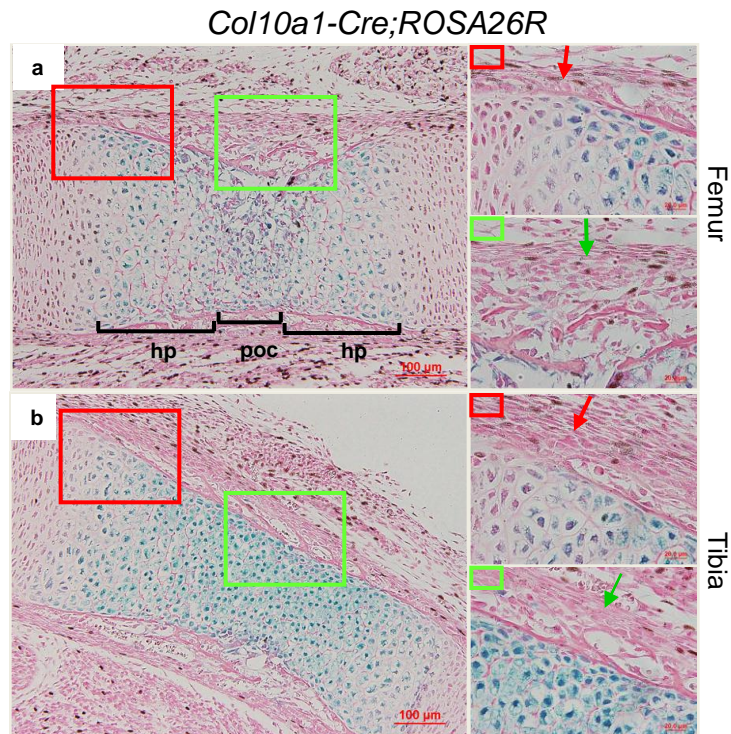

**C**

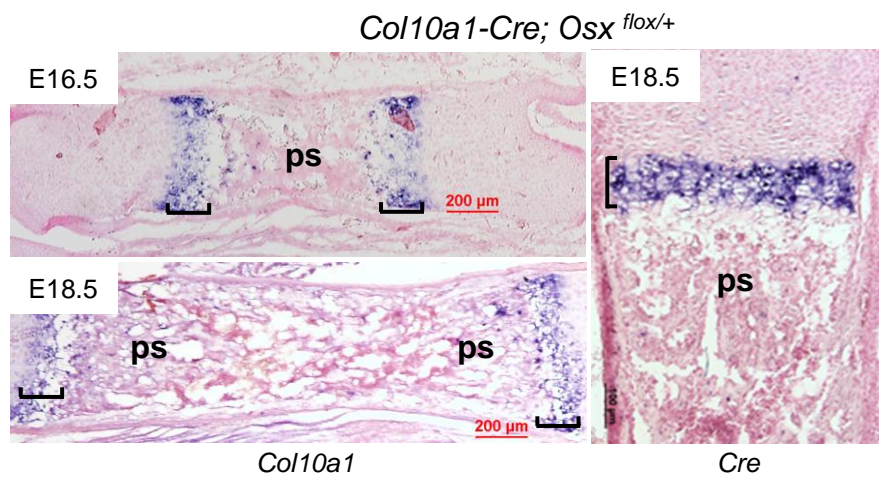

**D**

*Osx*<sup>flox/+</sup>

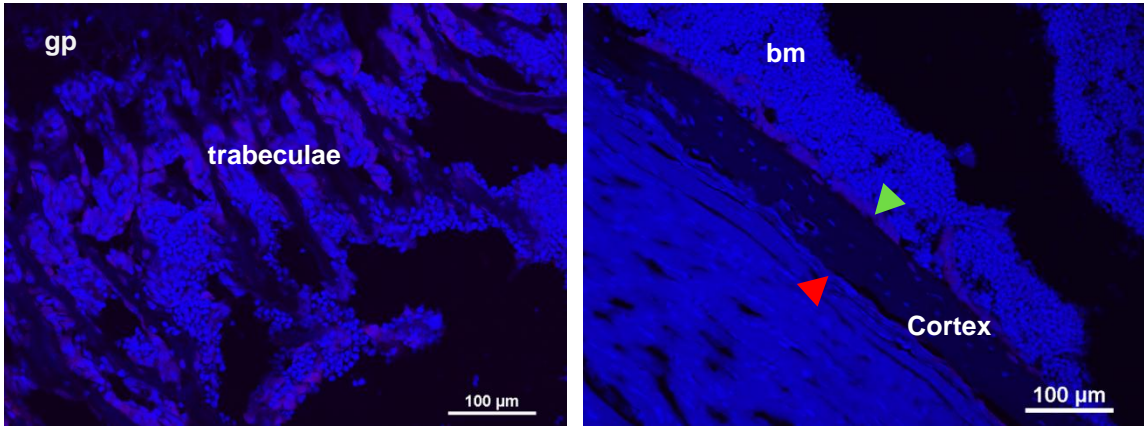

**E**

*Col10a1-Cre; Osx*<sup>flox/+</sup>

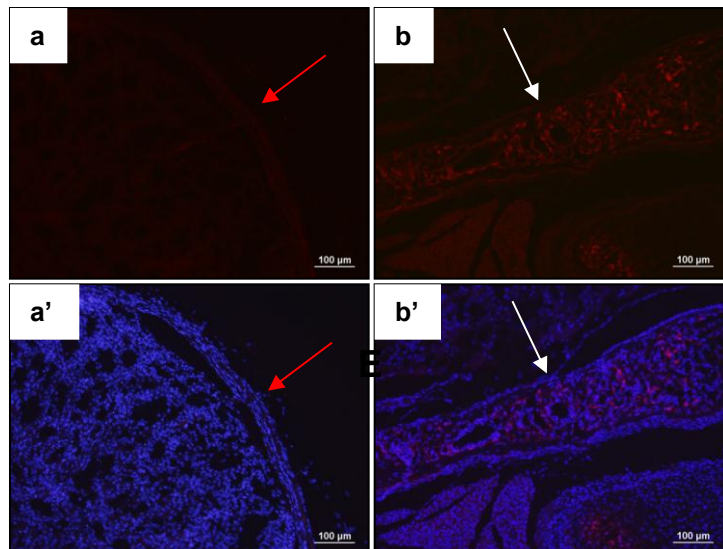

**F**

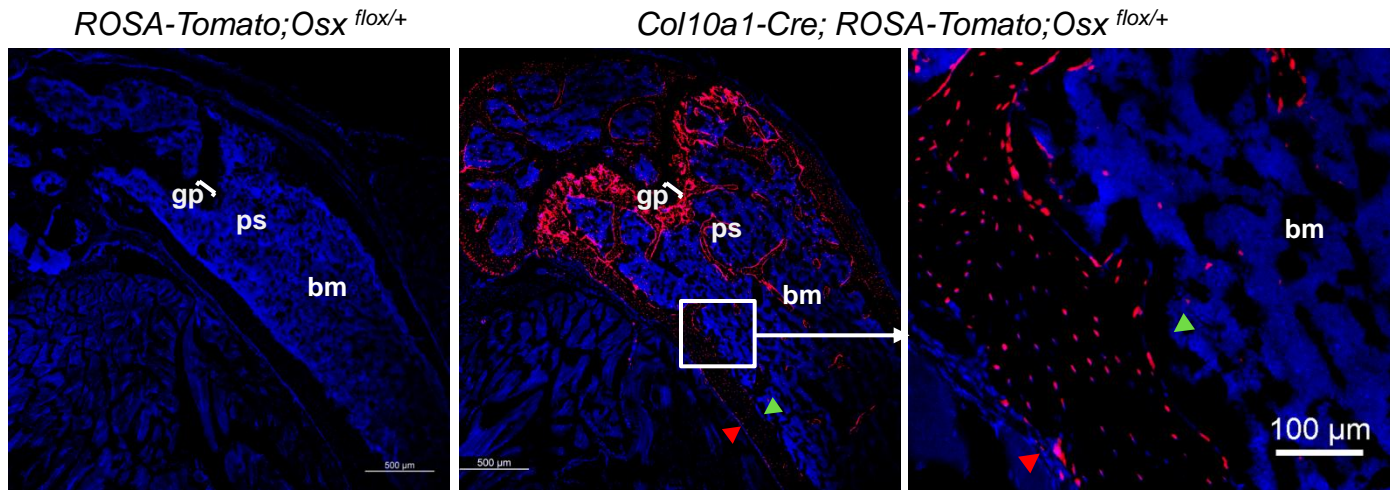

**G**

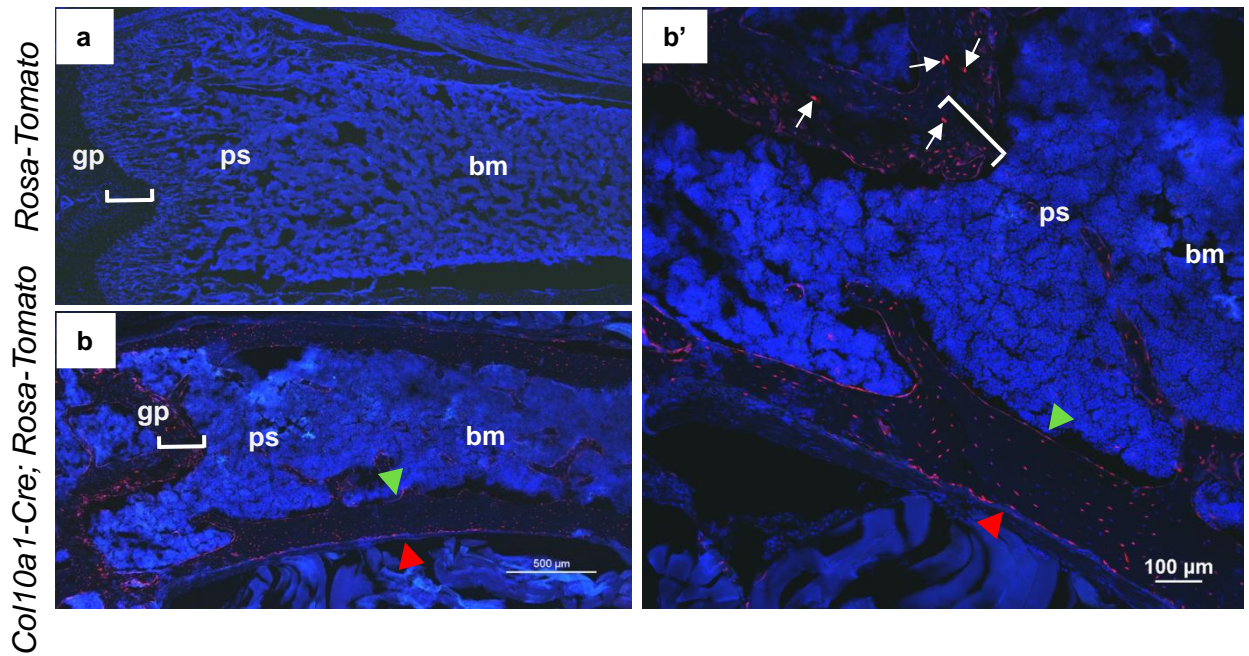

Supplement: Figure S1 — A: Illustration of Osx floxed allele and recombined Osx floxed allele. B: Panels a and b: LacZ staining of E15.5 Col10a1-Cre; ROSA26R hind limbs showed that Col10a1-Cre was active in hypertrophic chondrocytes, not in perichondrium (red arrows) or periosteum (green arrows). Panel a: femur; b: tibia. hp: hypertrophic chondrocytes; poc: primary ossification center. C: ISH revealed that there were practically no Col10a1-expressing cells in the primary spongiosa of the femurs of E16.5 and E18.5 Osxflox/+ embryos and there were no Cre-expressing cells in the primary spongiosa of the femur of a E18.5 Osxflox/+ embryo. Black brackets indicate hypertrophic zones; ps: primary spongiosa. D: Anti-EGFP IF showed that no EGFP+ cells were observed in the femur of a 2-week-old Osx flox/+ control mouse. Green arrowhead: endosteum; Red arrowhead: periosteum. E: Anti-EGFP IF showed that no EGFP+ cells were observed in the calvariae of E 18.5 Col10a1-Cre; Osxflox/+ embryo, while abundant EGFP+ cells were present in the basisphenoid bone of the same section. Panels a and a′: calvariae indicated by red arrows; b and b′: basisphenoid bone indicated by white arrows. Panels a and b: anti-EGFP (red); a′ and b′: anti-EGFP and DAPI (blue). F: The femur fluorescence images of 6-month-old Col10a1-Cre; ROSA-tdTomato;Osxflox/+ mice and ROSA-tdTomato;Osxflox/+ control mice. Red arrowhead: periosteum; Green arrowhead: endosteum; gp: growth plate (white brackets); ps: primary spongiosa; bm: bone marrow. G: The femur fluorescence images of 8-month-old Col10a1-Cre; ROSA-tdTomato (b and b′) and ROSA-tdTomato control mice (a). The white arrow indicates the bright Tomato+ cells in the growth plate of Col10a1-Cre; ROSA-tdTomato mouse. Red arrowhead: periosteum; Green arrowhead: endosteum; gp: growth plate (white brackets); ps: primary spongiosa; bm: bone marrow. (PDF) [file pgen.1004820.s001.pdf]

**A**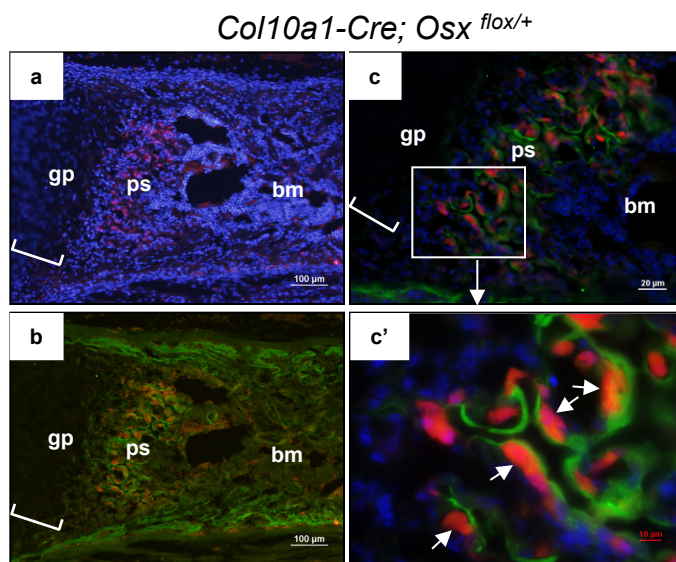**B**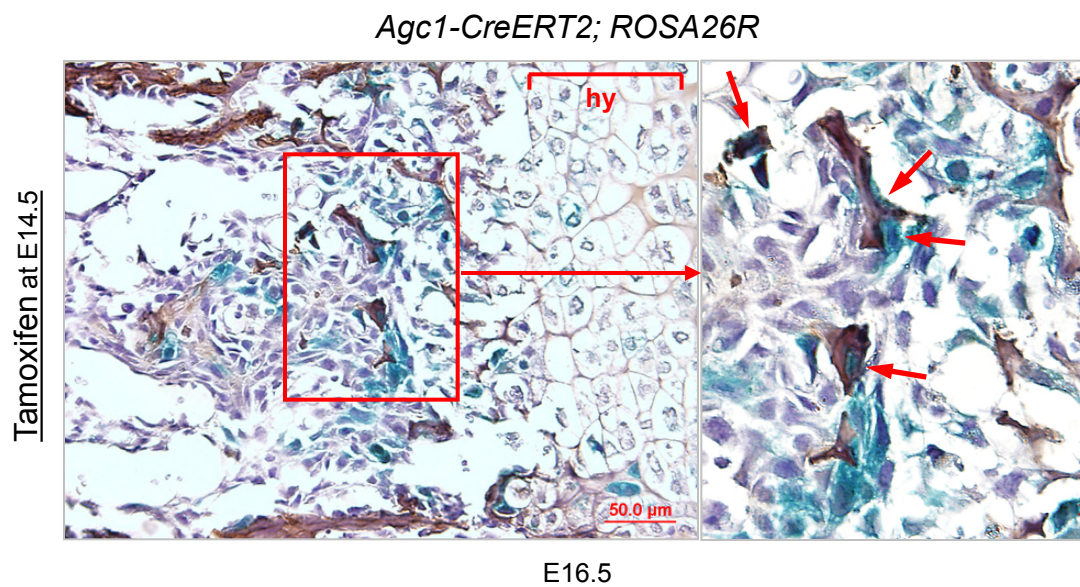

Supplement: Figure S3 — A: Double IF with anti-EGFP and anti-Col1a1 showed that EGFP+ (Osx−/+) cells (white arrows) were associated with Col1a1 in the femurs of E18.5 Col10a1-Cre; Osxflox/+ embryos. a: DAPI (blue) and EGFP (red); b: EGFP and Col1a1 (green); c & c′: DAPI, EGFP and Col1a1. White brackets: growth plate (gp); ps: primary spongiosa; bm: bone marrow. B: IHC with anti-BSP reveals that many of the LacZ+ cells (red arrows) in the primary spongiosa of the femur of a E16.5 Agc1-CreERT2; ROSA26R embryo were directly surrounded by BSP positive bone matrix (brown). hy: hypertrophic zone (red bracket). (PDF) [file pgen.1004820.s003.pdf]

*Agc1-CreERT2;2.3-gfp;Rosa-Tomato*

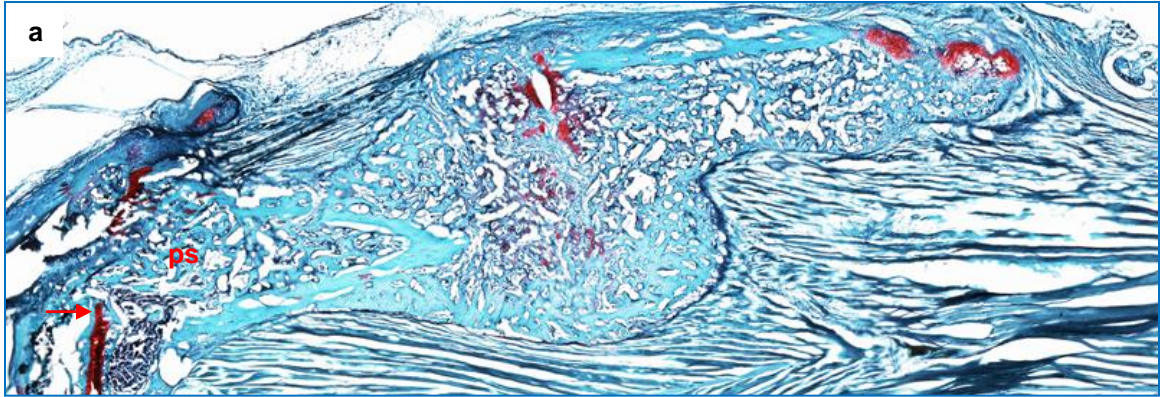

Tamoxifen at fracture day6/14

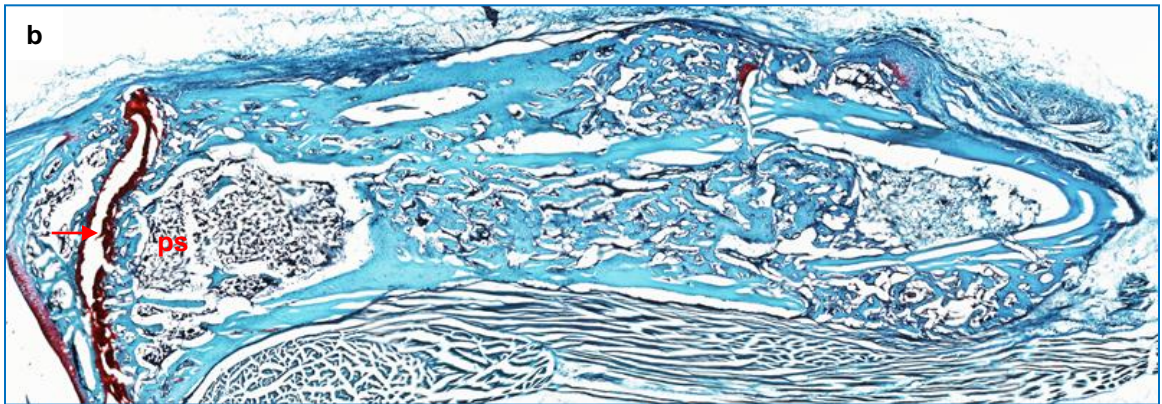

Tamoxifen at fracture day6/29

Supplement: Figure S5 — The images of Saf-O stained fractured tibia of 2.5-month-old Agc1-CreERT2;2.3-GFP;ROSA-tdTomato. Panel a: the mouse was injected with tamoxifen at 6 days after fracture surgery and was sacrificed 8 days after tamoxifen treatment. Panel b: the mouse was injected with tamoxifen at 6 days after fracture surgery and was sacrificed 23 days after tamoxifen treatment. Red arrow designates growth plate. (PDF) [file pgen.1004820.s005.pdf]
