# Supplementary material for: Chondrocytes Transdifferentiate into Osteoblasts in Endochondral Bone during Development, Postnatal Growth and Fracture Healing in Mice
Source: PLoS Genet. 2014 Dec 4;10(12):e1004820. doi: 10.1371/journal.pgen.1004820 (PMC4256265; doi:10.1371/journal.pgen.1004820)
Supplement: Figure S2 — A: LacZ staining of a E14.5 Agc1-CreERT2; ROSA26R embryos treated with tamoxifen at E13.5. Panels a, a′: E14.5 humerus; b, b′: tibia and fibula. The data show that Agc1-CreERT2 mediated recombination took place specifically in chondrocytes, not in perichondrium (red arrows). B: LacZ staining of a E13.5 Agc1-CreERT2; ROSA26R embryo treated with tamoxifen at E11. C: LacZ stained femur sections of Agc1-CreERT2; ROSA26R embryos, which were collected 9, 18 and 24 hours (E16.5) after tamoxifen injection at E15.5. Black brackets indicate hypertrophic zone and the black arrows designate the non-chondrocytic LacZ+ cells in primary spongiosa. D: LacZ stained femur (left panels) and tibia (right panels) sections of postnatal day 2 Agc1-CreERT2; ROSA26R mouse, born to a pregnant female treated with tamoxifen at E17.5. Primary ossification in femurs occurs earlier than in tibiae. The LacZ + cells in the primary spongiosa of femur (left panels) were likely derived from mature chondrocytes of completely established growth plates, whereas the LacZ+ cells in the primary spongiosa of tibiae (right panels) were likely derived from mature chondrocytes prior to growth plate formation. E: IF with anti-EGFP indicated that no EGFP+ cells were observed in the calvariae of 2-week-old Agc1-CreERT2; Osxflox/+ mouse, an offspring of female treated with tamoxifen at E14.5, while abundant EGFP+ cells were present in the basisphenoid of the same section. Panels a and a′: calvariae between the red arrows; b and b′: basisphenoid designated by white arrows. Panels a and b: anti-EGFP (red); a′ and b′: anti-EGFP and DAPI (blue). gp: growth plate (white brackets). (PDF) [file pgen.1004820.s002.pdf]

A

Agc1-CreERT2; ROSA26R

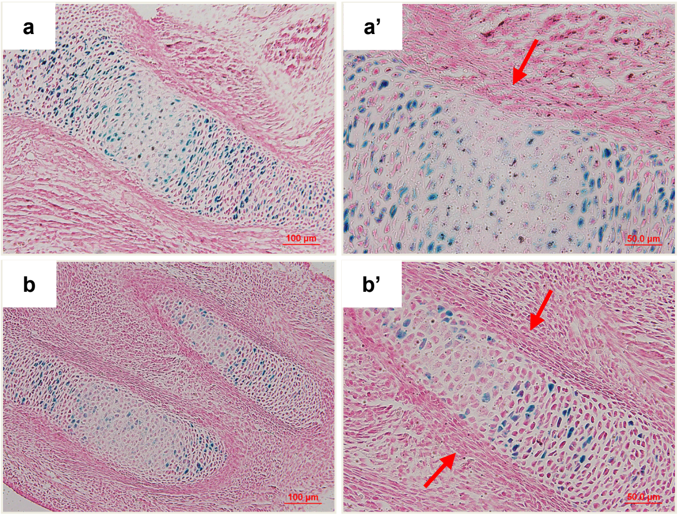

Tamoxifen at E13.5

B

ROSA26R *Agc1-CreERT2; ROSA26R*

E13.5

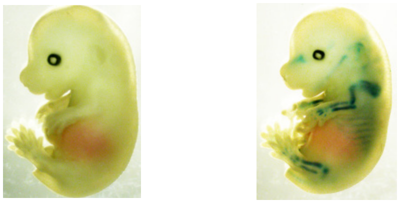

Tamoxifen at E11

C

Tamoxifen at E15.5

Agc1-CreERT2; ROSA26R

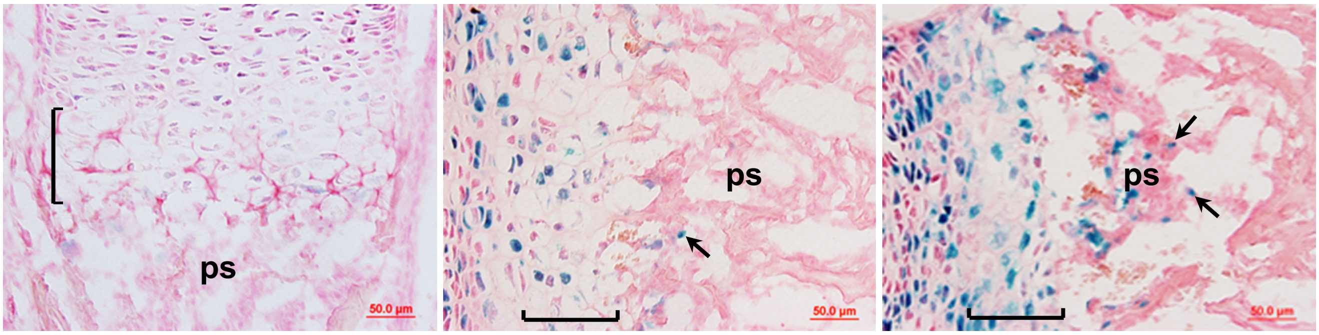

9hrs-fe 18hrs-fe 24hrs-fe/E16.5

Zhou_Supplement Fig 2.

D

Tamoxifen at E17.5/Day 2

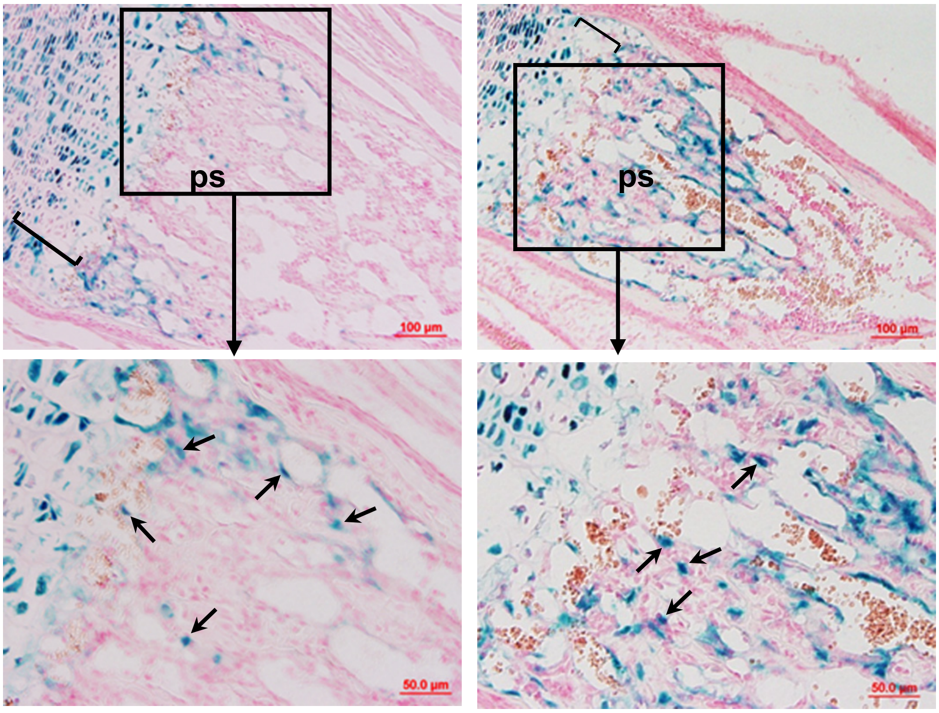

Fe **Tib**

E

Agc1-CreERT2; Osx $^{flox/+}$

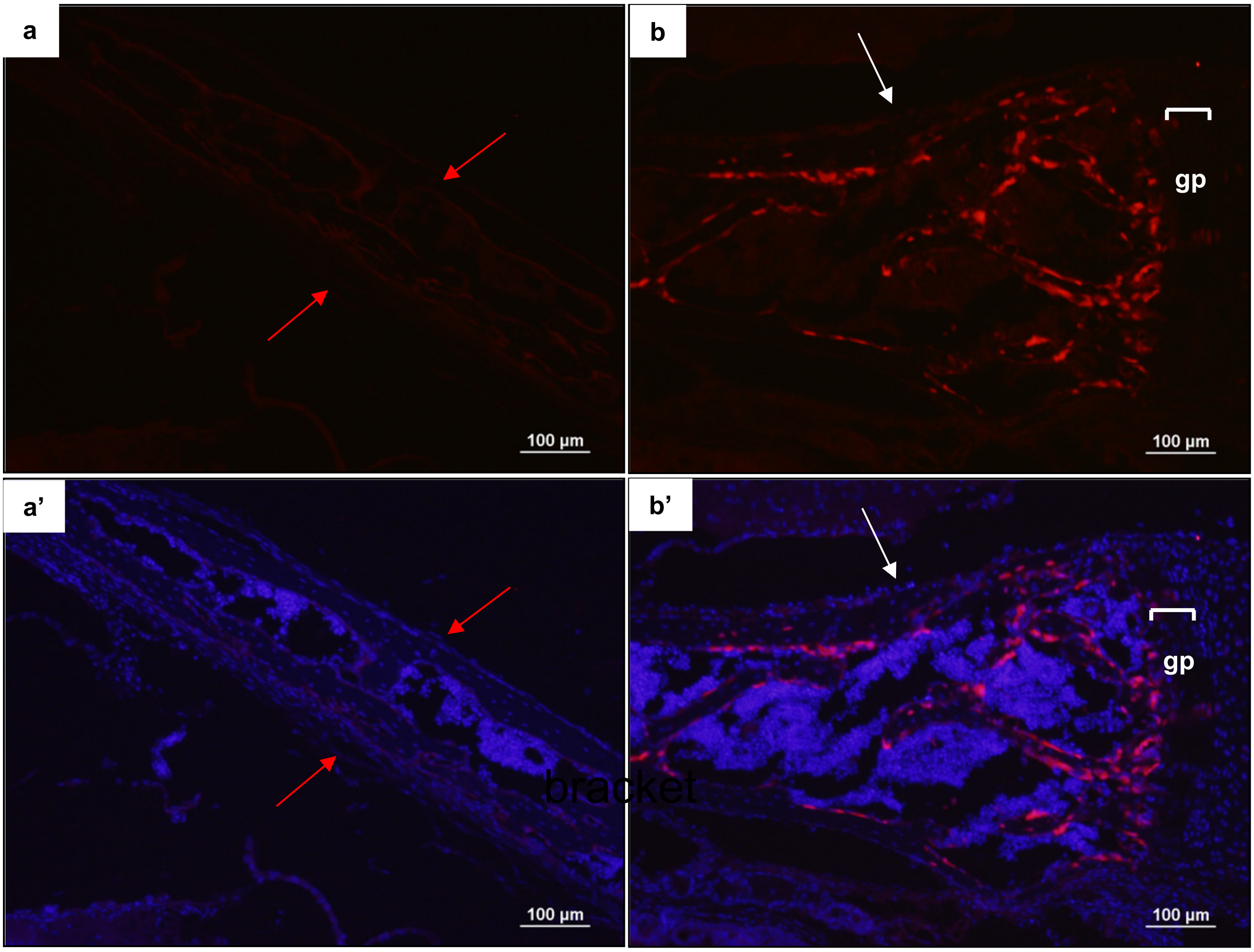

2 week
