# Supplementary material for: Chondrocytes Transdifferentiate into Osteoblasts in Endochondral Bone during Development, Postnatal Growth and Fracture Healing in Mice
Source: PLoS Genet. 2014 Dec 4;10(12):e1004820. doi: 10.1371/journal.pgen.1004820 (PMC4256265; doi:10.1371/journal.pgen.1004820)

Zhou_Supplement Fig 4.

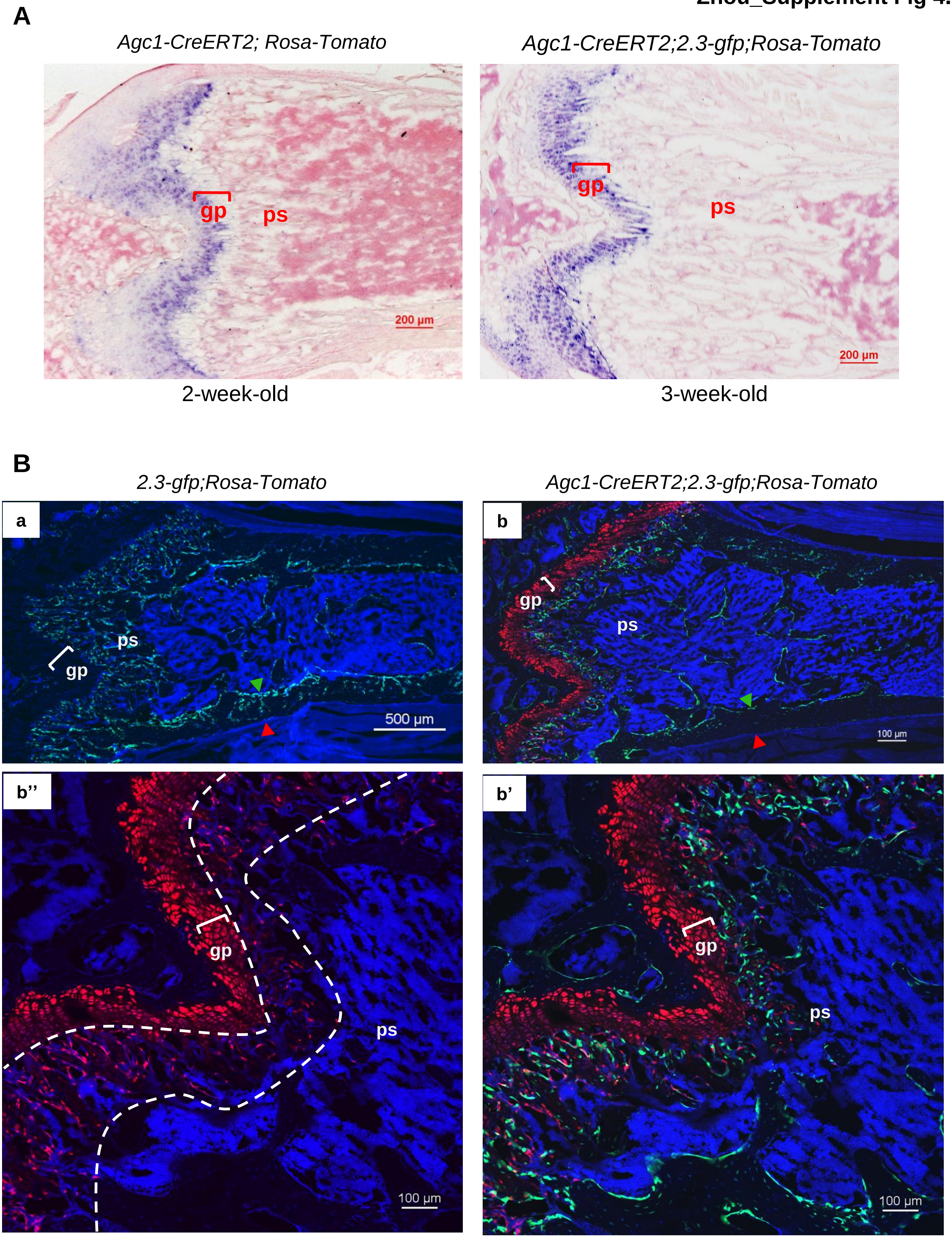

Supplement: Figure S4 — A: Agc1 ISH of femurs of 2-week-old Agc1-CreERT2;ROSA-tdTomato (left) and 3-week-old Agc1-CreERT2; 2.3-GFP;ROSA-tdTomato (right) mice. B: The fluorescence images of femur sections of 13-week-old 2.3-GFP;ROSA-tdTomato (a) and Agc1-CreERT2;2.3-GFP;ROSA-tdTomato (b, b′ and b″).mice, which were treated with tamoxifen at 11 weeks. The Tomato+ cell in the primary spongiosa were distributed in the area outlined by the white dotted lines. (PDF) [file pgen.1004820.s004.pdf]
