# Supplementary material for: Chondrocytes Transdifferentiate into Osteoblasts in Endochondral Bone during Development, Postnatal Growth and Fracture Healing in Mice
Source: PLoS Genet. 2014 Dec 4;10(12):e1004820. doi: 10.1371/journal.pgen.1004820 (PMC4256265; doi:10.1371/journal.pgen.1004820)
Supplement: Table S1 — Dynamics of CreERT2 inducibility by tamoxifen during the skeletal development of Agc1-CreERT2;ROSA26R embryos. (PDF) [file pgen.1004820.s006.pdf]

Suppl. Table 1

Tamoxifen injection (embryonic days)	x-gal staining (embryonic days)	x-gal staining Results
11.5	12.5	negative
8.5	13.5	negative
9.5	13.5	negative
11	13.5	positive
