# Supplementary material for: Chondrocytes Transdifferentiate into Osteoblasts in Endochondral Bone during Development, Postnatal Growth and Fracture Healing in Mice
Source: PLoS Genet. 2014 Dec 4;10(12):e1004820. doi: 10.1371/journal.pgen.1004820 (PMC4256265; doi:10.1371/journal.pgen.1004820)
Supplement: Table S2 — Estimation of the percent of mature osteoblasts (Ocn+) that are derived from chondrocytes (EGFP+Ocn+) in the trabecular and endosteal regions of 1-month-old Col10a1-Cre;Osxflox/+ mice. The 20× images of anti-Ocn and anti-GFP stained femur sections were used for counting. For the trabecular region, the total numbers of EGFP+Ocn+ (or Ocn+) cells for each sample were the sums of EGFP+Ocn+ (or Ocn+) cells from 4 overlapping images covering the trabecuar region (n = 4). For the endosteal region, the total numbers of EGFP+Ocn+ (or Ocn+) cells for each sample were the sums of EGFP+Ocn+ (or Ocn+) cells from 4 overlapping cortical images (n = 3). NIS-Elements AR software program was used for counting. (PDF) [file pgen.1004820.s007.pdf]

Suppl. Table 2

Trabecular region				Endosteum surface			
Sample#	Ocn^+ cell#	GFP^+Ocn^+ cell#	GFP^+Ocn^+/Ocn^+ cell %	Sample#	Ocn^+ cell#	GFP^+Ocn^+ cell#	GFP^+Ocn^+/Ocn^+ cell %
1	35	25	71.4	1	65	33	50.8
2	96	49	51.0	2	90	59	65.5
3	41	24	58.5	3	34	21	61.8
4	75	47	62.7				
**Avg.±SD %**			**63±7.3**	**Avg.±SD %**			**62±6.5**
