# Supplementary material for: Chondrocytes Transdifferentiate into Osteoblasts in Endochondral Bone during Development, Postnatal Growth and Fracture Healing in Mice
Source: PLoS Genet. 2014 Dec 4;10(12):e1004820. doi: 10.1371/journal.pgen.1004820 (PMC4256265; doi:10.1371/journal.pgen.1004820)
Supplement: Table S3 — Estimation of the percentage of osteoblasts (EGFP+) that are derived from chondrocytes (Tomato+EGFP+) in the trabecular and endosteal regions of 3-week-old Col10a1-Cre;2.3Col1-GFP;ROSA-tdTomato triple transgenic mice. The numbers of EGFP+ cells and Tomato+EGFP+ cells were obtained using the same method described in Table S2 (n = 5). (PDF) [file pgen.1004820.s008.pdf]

Suppl. Table 3

Trabecular region				Endosteum surface			
Sample#	GFP^+ cell#	GFP^+Tm^+ cell#	GFP^+Tm^+/GFP^+ cell %	Sample#	GFP^+ cell#	GFP^+Tm^+ cell#	GFP^+Tm^+/GFP^+ cell %
1	225	132	58.7	1	126	83	65.9
2	266	154	57.9	2	125	75	60.0
3	263	149	56.7	3	140	101	72.1
4	271	187	69.0	4	201	129	64.2
5	338	202	59.7	5	195	132	67.7
**Avg.±SD %**			**60.4±4.8**	**Avg.±SD %**			**68.0±4.1**
